# Second-generation piperazine derivatives as promising radiation countermeasures†

**DOI:** 10.1039/d4md00311j

**Published:** 2024-07-11

**Authors:** Vojtěch Chmil, Natálie Živná, Marcela Milanová, Alžběta Filipová, Jaroslav Pejchal, Lukáš Prchal, Darina Muthná, Vít Řeháček, Martina Řezáčová, Jan Marek, Aleš Tichý, Radim Havelek

**Affiliations:** a Department of Radiobiology, Military Faculty of Medicine, University of Defence in Brno Trebesska 1575 500 05 Hradec Kralove Czech Republic; b Department of Toxicology and Military Pharmacy, Military Faculty of Medicine, University of Defence in Brno Trebesska 1575 500 05 Hradec Kralove Czech Republic; c Biomedical Research Centre, University Hospital Hradec Kralove Sokolska 581 500 05 Hradec Kralove Czech Republic; d Department of Medical Biochemistry, Faculty of Medicine in Hradec Kralove, Charles University Simkova 870 500 03 Hradec Kralove Czech Republic havelekr@lfhk.cuni.cz; e Transfusion Department, University Hospital Hradec Kralove Sokolska 581 500 05 Hradec Kralove – Novy Hradec Kralove Czech Republic; f Department of Epidemiology, Military Faculty of Medicine, University of Defence in Brno Trebesska 1575 500 05 Hradec Kralove Czech Republic

## Abstract

The increasing threat of nuclear incidents and the widespread use of ionizing radiation (IR) in medical treatments underscore the urgent need for effective radiation countermeasures. Despite the availability of compounds such as amifostine, their clinical utility is significantly limited by adverse side effects and logistical challenges in administration. This study focuses on the synthesis and evaluation of novel piperazine derivatives as potential radioprotective agents, with the aim of overcoming the limitations associated with current countermeasures. We designed, synthesized, and evaluated a series of 1-(2-hydroxyethyl)piperazine derivatives. The compounds were assessed for cytotoxicity across a panel of human cell lines, and for their radioprotective effects in the MOLT-4 lymphoblastic leukemia cell line and in peripheral blood mononuclear cells (PBMCs) exposed to gamma radiation. The radioprotective efficacy was further quantified using the dicentric chromosome assay (DCA) to measure DNA damage mitigation. Among the synthesized derivatives, compound 6 demonstrated the most significant radioprotective effects *in vitro*, with minimal cytotoxicity across the tested cell lines. Compound 3 also showed notable efficacy, particularly in reducing dicentric chromosomes, thus indicating its potential to mitigate DNA damage from IR. Both compounds exhibited superior safety profiles and effectiveness compared to amifostine, suggesting their potential as more viable radioprotective agents. This study highlights the development of novel piperazine derivatives with promising radioprotective properties. Compound 6 emerged as the leading candidate, offering an optimal balance between efficacy and safety, with compound 3 also displaying significant potential. These findings support the further development and clinical evaluation of these compounds as safer, and more effective radiation countermeasures.

## Abstract 2

The increasing threat of nuclear incidents and the widespread use of ionizing radiation (IR) in medical treatments underscore the urgent need for effective radiation countermeasures.

## Graphical abstract

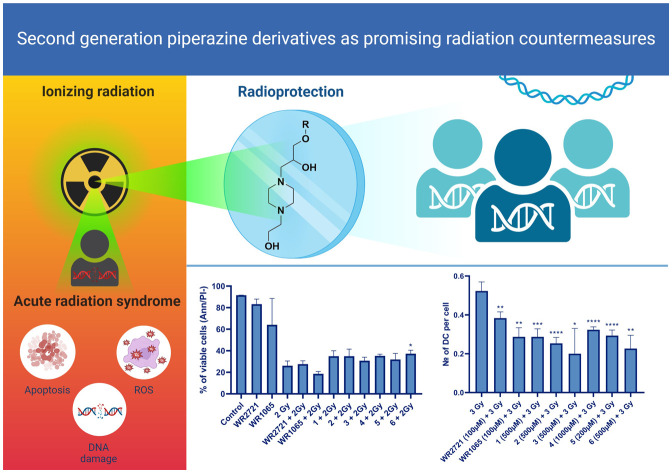

## Introduction

In light of the prevailing geopolitical situation, the world faces an escalating risk of nuclear conflict greater than ever before. A pivotal consequence of nuclear weaponry is the emission of IR, for which our protective capacities remain notably limited.^1–3^ Moreover, millions of patients undergo exposure to IR annually in medical facilities. These facts underscore the critical necessity for advancing the development of effective and safe radiation countermeasures.^4–6^

IR causes detrimental consequences in normal healthy tissues. At the organ level, exposure to IR results in the demise of crucial cell populations, such as bone marrow hematopoietic stem cells or enterocytes. Cell death is triggered by DNA damage, which activates signaling pathways, and inflammatory responses. These cascading events prompt cell death or senescence in organs, contributing to the radiotoxicity. The consequences are particularly notable in tissues undergoing radiotherapy for cancer treatment, where the desired apoptosis of cancer cells inadvertently leads to damage in adjacent normal tissues.^7–9^

At the molecular level, IR initiates intricate cellular responses, notably by activating programmed cell death pathways. Two major signaling pathways, extrinsic and intrinsic, are recognized, with specific cytokines and factors such as tumor necrosis factor alpha (TNF-α) or fas ligand playing key roles. The intrinsic pathway responds to various stimuli, including growth factor deprivation, oxidants, DNA damaging agents, or microtubule targeting drugs. The molecule p53-upregulated mediator of apoptosis (PUMA) emerges as a central figure in radiation-induced cell death signaling. Identifying PUMA as a crucial target both for radioprotection and for the treatment of neurodegenerative and cardiovascular diseases highlights the intricate connection between IR, molecular responses, and the potential for precise interventions at the molecular level.^10,11^

Current radiation countermeasures are limited by either limited effectiveness or excessive toxicity, leading to significant adverse effects upon use.^12^ Amifostine, a well-studied radioprotective agent, illustrates this dilemma. While it offers the highest known efficacy in IR protection, its application is hindered by severe undesired effects, including hypotension, nausea, and vomiting. The compound's high toxicity necessitates caution, especially at the doses required for optimal protection. Moreover, its narrow administration window, around 30 min before radiation exposure, poses logistical challenges, particularly in emergency situations. The primarily intravenous route of administration further complicates its widespread use.^13,14^

While amifostine has received Food and Drug Administration (FDA) approval for specific clinical indications, including the reduction of cumulative renal toxicity associated with cisplatin and the management of xerostomia during radiotherapy for head and neck cancers, its use as a general radioprotector remains unapproved. Various attempts have been made to address its toxicity issues, such as modification of dosage regimens and exploring alternative delivery methods. However, none of these strategies has completely alleviated the concerns surrounding its side effects. Recent investigations into amifostine's potential as a radioprotector for acute radiation syndrome (ARS) have shown promise but have not yet resulted in FDA approval for non-clinical use. The challenges in reducing its side effects while maintaining optimal efficacy highlight the need for continued research and development, exploring avenues such as poly-pharmacy approaches and alternative combinations to achieve synergistic effects.^12,15,16^ In our study, we selected amifostine (WR2721) and its active metabolite WR1065 as comparators for the newly designed molecules.

The study conducted by Mustata *et al.*^17^ identified compounds that demonstrated considerable protection against PUMA-dependent and radiation-induced apoptosis. One of those compound series was derivatives of 1-(2-hydroxyethyl)piperazine, which were subsequently further optimized by our group.^18,19^ We developed several molecules with pronounced radioprotective effects evaluated *in vitro* and in a murine model *in vivo*. Our current research aims to advance promising molecules, building on the previous studies to achieve a more efficient structural design. Leveraging insights from its radioprotective properties, we systematically design and synthesize additional derivatives of this structure. By optimizing the chemical structure based on the acquired knowledge, we aim to enhance the radioprotective potential and reduce toxicity both *in vitro* and *in vivo*.

Based on the optimization process of the aforementioned molecules, we synthesized eight compounds. We assessed their toxicity *in vitro* using a panel of human cell lines from eight different tissue origins, and PBMCs. The integration of toxicity assessments conducted on human cell lines and PBMCs aided in identifying the most promising derivatives for further investigation of their radioprotective activity. Given the established understanding that IR disrupts hematopoiesis, we assessed the potential radioprotective effects on the MOLT-4 lymphoblastic leukemia cell line of hematological histotype origin and on the PBMCs model.

## Results and discussion

### Design and synthesis of innovative piperazine derivatives for enhanced radioprotection

In the study of Mustata *et al.*,^17^ the *in silico* screening of the ZINC 8.0 database yielded 142 compounds. Twenty of those compounds were extracted based on ADME/T calculations and most of them contained a 1-(2-hydroxyethylpiperazine) moiety. This molecular component seems to be crucial for the compound's radioprotective effect. Despite it being several years since the original publication, the scientific group has not published any research since. However, the published results do show that even slight modifications to the structure result in a different outcome. That can be seen in our previous studies. Some piperazine derivatives cause radiosensitisation, while other resulted in radioprotection.^19^

Compound CLZ-8; another piperazine derivative; is highly lipophilic due to the moiety connected to the hydrophilic piperazine and exhibits good radioprotective properties *in vitro*, however, authors needed to use rather low concentrations (up to 5 μM) in comparison to our working range of concentrations.^20^ Albeit, *in vivo*, CLZ-8 was used in 200 mg kg^−1^ dose for mice and significantly improved their survival, though CLZ-8 inflicted damage was not explored by the authors. Another piperazine derivative, 1-(4-nitrobenzenesulfonyl)-4-penylpiperazine, inhibited apoptosis.^21^ The structure was successful in protecting the gastrointestinal system, but the mechanism is not well explored. Although, a small piperazine derivative, diethylcarbamazine, protected from radiation by inhibiting NFκB and COX-2, but as of yet, it is unclear if the mechanism is applicable on our derivatives.^22^ As such, designing and evaluating novel structures advances the field of radiation countermeasures.

From our previous studies,^18^ compound 1 named 1-(4-(2-hydroxyethyl)piperazin-1-yl)-3-(2-methoxy-4-nitrophenoxy)propan-2-ol was determined as the most effective of the series, and therefore serves as the leading and comparative compound in this study. Compound 2 is an inversion of the original compound 1, with the phenoxy substituents exchanging positions. In compounds 3 and 5 the methoxy group was discarded, and the nitro-group was preserved in combination with halogen substituent/s. The halogen atoms were introduced into the structure to increase the compound's lipophilicity, and thus improve its permeability into the cells.^23,24^ Subsequently the NO_2_ group was also abandoned and only halogen substituents were implemented into the structure (4, 6, 7, and 8), with substance 7 being further enlarged with an extra aromatic ring.

**Fig. 1:**
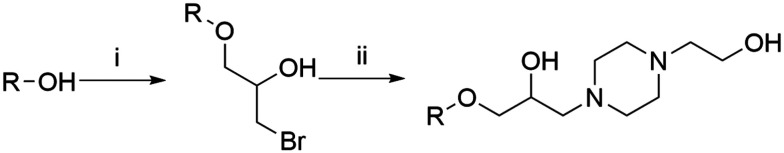
Synthetic pathway towards 1-(4-(2-hydroxyethyl)piperazin-1-yl)-3-phenoxypropan-2-ol derivatives (1–8). Reagents and conditions: (i) epibromohydrin, 135 °C, 2 h, catalytic amount of piperidine; F 1-(2-hydroxyethyl)piperazine, K_2_CO_3_, in acetonitrile, 85 °C, 4 h.

The general synthetic procedure followed Fig. 1. In the first step, substituted aromatic alcohols serve as nucleophiles, their nucleophilic properties being further potentiated by the presence of piperidine.^25^ Epibromohydrins – electrophilic compounds – offer high synthetic potential due to the tension of the three-membered ring and the presence of three electrophilic carbons.^26^ The main purpose of the purification is to remove the excess epibromohydrin while achieving reasonable purity of the resulting precursors. In the second step, 1-(2-hydroxyethyl)piperazine serves as a nucleophile, enhanced in the presence of K_2_CO_3_. The reaction mixture is purified using column chromatography to acquire the desired substances in purity 95% or higher.

The identity and purity of the substances were verified by nuclear magnetic resonance (NMR) and high-resolution mass spectrometry (HRMS) analysis. MarvinSketch 14.9.8.0 software was used to obtain calculated log *P* (clog *P*) values. Lower clog *P* values are exhibited by substances (1–4), containing a NO_2_ group, halogen atom, methoxy group, or their respective combinations. The remaining compounds 5–8, which possess extra halogens or an extra aromatic ring, express higher clog *P* (Table 1).

**Table 1:** List of the tested substances indicating the substituted aromatic attachments (R) of the molecule 1-(4-(2-hydroxyethyl)piperazin-1-yl)-3-phenoxypropan-2-ol and their clog *P* values

Compound	R	clog *P*
1	2-Methoxy-4-nitrophenyl	0.143
2	4-Methoxy-2-nitrophenyl	0.143
3	2-Fluoro-4-nitrophenyl	0.444
4	4-Fluorophenyl	0.504
5	4-Chloro-3-nitrophenyl	0.905
6	3-Chlorophenyl	0.965
7	4-(4-Fluorophenoxy)phenyl	2.004
8	2,4,5-Trichlorophenyl	2.173

However, an increase in log *P* values can and usually does result in higher toxicity of the compounds, and therefore, a compromise needs to be reached between optimal cell permeability, safety profile, and solubility.^27^ As can be seen in the *in vitro* cytotoxicity evaluation, compounds 7 and 8 exhibit severe toxicity to most of the tested cell lines, almost comparable to that of amifostine itself. Their clog *P* value is the highest of the prepared compounds. Compound 7 contains an additional aromatic ring, which seems to be detrimental to the compound's safety. Compound 8 containing three chlorine atoms is safer than compound 7, but is still unacceptably toxic. The design of this compound could be improved by switching the chlorine atoms for the more polar fluorine, since halogens do appear to be the superior substituents. These findings assure the unique place of halogens in the development of future radioprotective molecules, already explored in earlier studies.^19^ Compounds with clog *P* up to 1 appear to be safe.

Compound 6 performed the best radioprotective effect, and it also presented the highest clog *P* of the tested cohort. That could mean that a reasonably high log *P* value might be desirable.^28^ However, the differences between the compounds are marginal and all provided significant radioprotection in comparison to both amifostine and its active metabolite. The weakest results were exhibited by 3 and 5, which both contain a halogen atom in combination with a NO_2_ group, suggesting that this variation might not be optimal. However, in respect of the number of dicentric chromosomes, compound 3 performed the best, and hence the combination of NO_2_ group and halogens might still be useful. Compound 6 also showed very good results in dicentric chromosome assay, which further prompts that a reasonable increase in lipophilicity might bear fruit.

### *In vitro* cytotoxicity evaluation

**Fig. 2:**
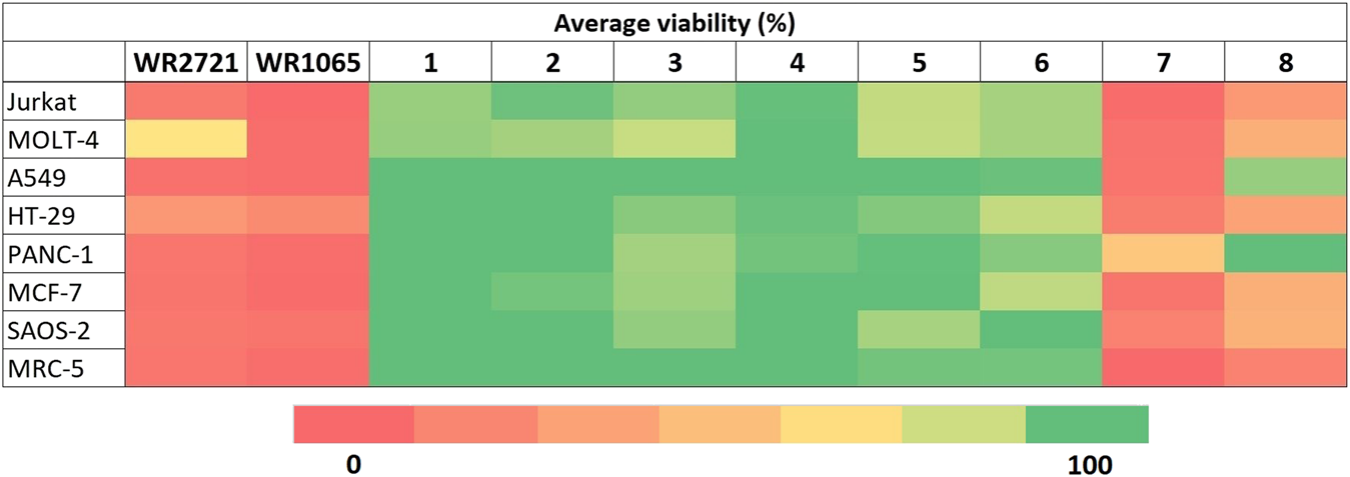
Proliferation of individual cell lines exposed to the compounds at 100 μM for 48 h.

**Fig. 3:**
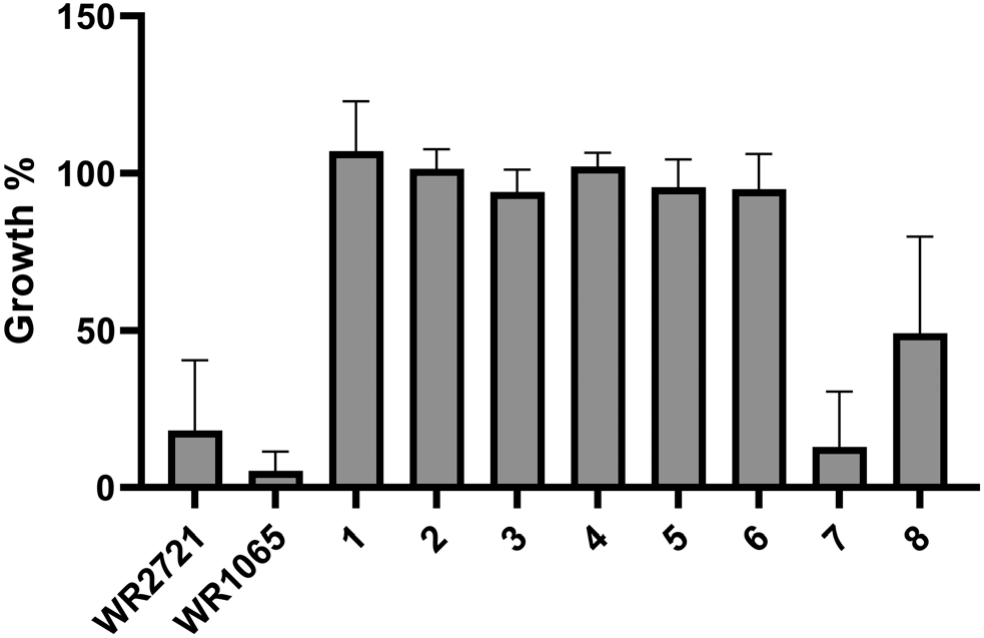
Overall cytotoxic effect of compounds expressed as growth percent – the mean value of the proliferation of individual cell lines treated at 100 μM for 48 h. Error bars represent the standard errors of the mean.

A thorough analysis of antiproliferative activity was performed across various cell culture types representing different histotypes: leukemia (Jurkat, MOLT-4), lung adenocarcinoma (A549), colorectal adenocarcinoma (HT-29), pancreatic carcinoma (PANC-1), breast adenocarcinoma (MCF-7), osteosarcoma (SAOS-2), and one non-cancerous cell line (fetal lung fibroblasts MRC-5). The cell lines were exposed to the newly synthesized compounds at a concentration of 100 μM for 48 h in a single-dose treatment. Changes in cell proliferation compared to the vehicle 0.1% dimethyl sulfoxide (DMSO) control were determined using the WST-1 assay, which is based on mitochondrial dehydrogenase metabolic activities. All data are an average of at least three independent measurements, Fig. 2 displays the results in a clear heatmap, with complete data provided in the ESI† (Fig. S1). The overall cytotoxic activity of each compound was expressed as growth percent (GP) – the mean of the proliferation of all cell lines treated with the same compound. The inhibitory rates obtained indicated that two compounds (7 and 8) exhibited stand-alone cytotoxicity and were therefore excluded from subsequent experiments exploring *ex vivo* cytotoxicity and the radioprotective effect (Fig. 3).

### *Ex vivo* cytotoxicity evaluation

**Fig. 4:**
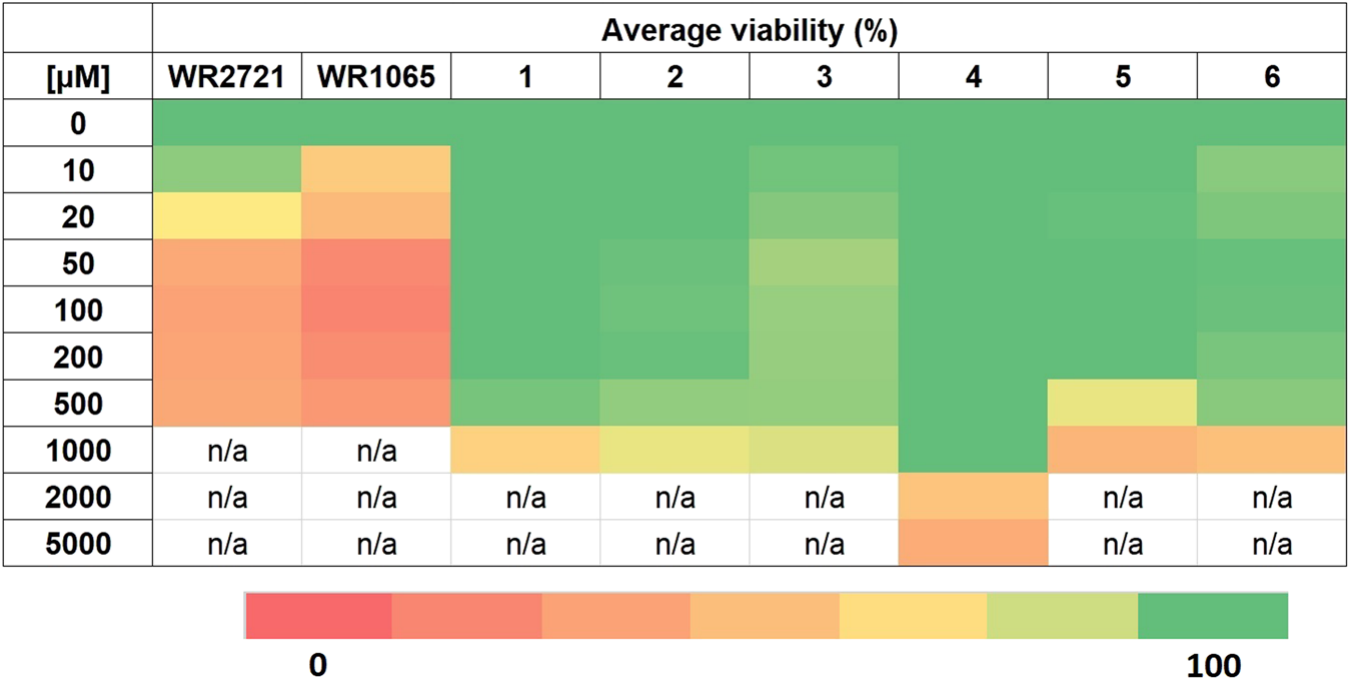
Viability of PBMCs after 48 h exposure to the tested compounds. The viability of untreated control is 100%.

Based on the cytotoxicity screening on the cell lines, compounds 1–6, along with amifostine and its active form WR1065, were selected for toxicity assessment on PBMCs using the MTS colorimetric assay. The results revealed that WR1065 exhibited the highest toxicity, with the lowest tested concentration of 10 μM causing a 43% decrease in cell viability. Amifostine (WR2721) followed, showing a significant decrease at a concentration of 20 μM. All six new compounds demonstrated lower toxicity. The data for all screened compounds are presented in a form of a heatmap in Fig. 4. The values are an average of at least three independent measurements and complete data are enclosed in ESI† (Fig. S2). To represent the overall inhibitory activity of each compound, viability was calculated as the mean of at least three independent measurements. The purpose was to determine the optimal non-toxic concentration for subsequent use in radioprotection tests. For each substance, the concentration at which the viability of PBMCs did not drop below 100% (control group) at the 0.05 significance level and was at the same time higher than 90% was determined. For compound 4 this concentration was 1 mM; for compounds 1, 2, 3, and 6 the concentration was 500 μM; for 5 it was 200 μM; for WR2721 it was 10 μM; and WR1065 was toxic even at the lowest tested concentration of 10 μM.

### Effect of ionizing radiation on the viability of MOLT-4 cells stand-alone and in combination with inhibitor pretreatment

**Fig. 5:**
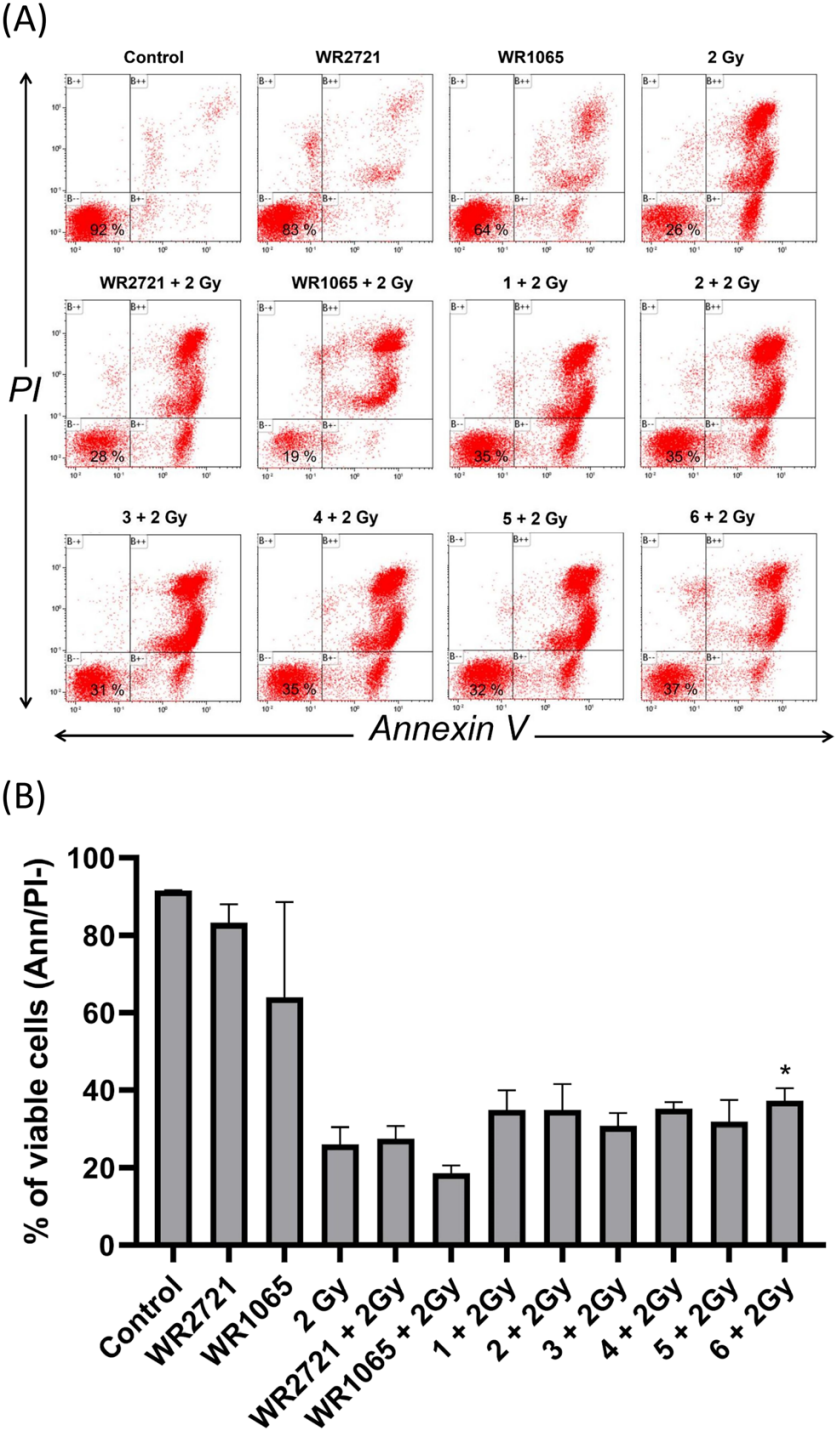
Flow cytometric quantification of MOLT-4 cell viability. Cell viability was determined with annexin V/PI staining. MOLT-4 cells were exposed to IR (2 Gy) alone or after 2 h pretreatment with the compounds at a concentration of 100 μM for the incubation interval of 24 h and then subjected to flow cytometry analysis to determine the population of viable (annexin V/PI negative) cells. (A) The top panels consist of flow cytometry histograms illustrating the percentage of viable cells, representing one of the representative experiments that were independently repeated at least three times. (B) The column graph shows percentages of viable cells. The asterisk above the individual bars represents statistical significance (*P* < 0.05) related to 2 Gy. Cells treated with 0.1% DMSO were used as the negative vehicle treatment control.

**Fig. 6:**
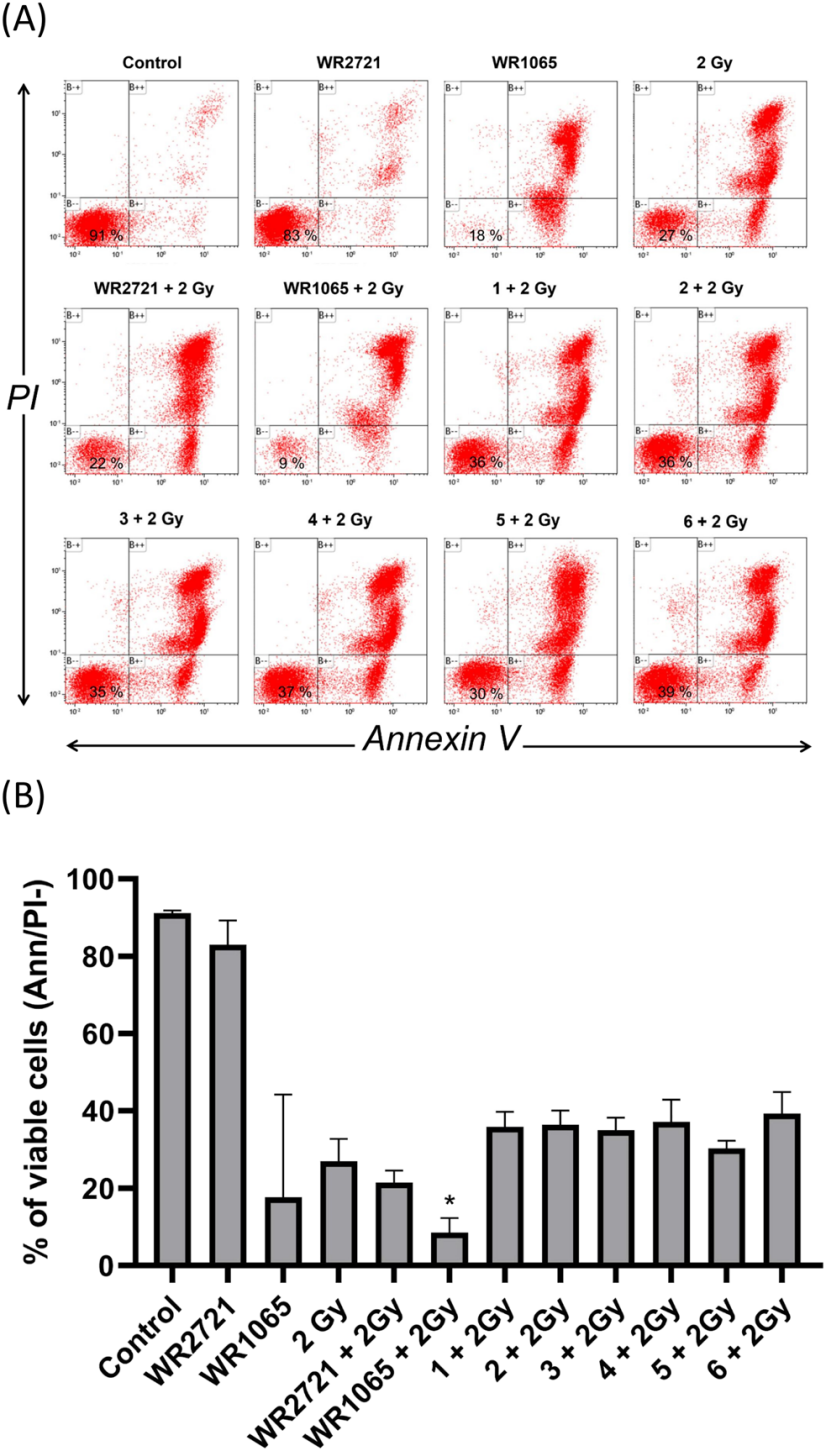
Flow cytometric quantification of MOLT-4 cell viability. Cell viability was determined with annexin V/PI staining. MOLT-4 cells were exposed to IR (2 Gy) alone or after 2 h pretreatment with the compounds at a concentration of 200 μM for the incubation interval of 24 h and then subjected to flow cytometry analysis to determine the population of viable (annexin V/PI negative) cells. (A) The top panels consist of flow cytometry histograms illustrating the percentage of viable cells, representing one of the representative experiments that were independently repeated at least three times. (B) The column graph shows percentages of viable cells. The asterisk above the individual bars represents statistical significance (*P* < 0.05) related to 2 Gy. Cells treated with 0.1% DMSO were used as the negative vehicle treatment control.

Initial findings indicated that cytotoxicity screening with a 100 μM dose of compounds 1–6 revealed no significant reduction in proliferation across multiple cell histotypes. Consequently, we investigated whether the pre-application of these substances can augment the survival of MOLT-4 cells after irradiation exposure. The radioprotective investigation utilized a leukemia MOLT-4 cell model of hematopoietic origin with flow cytometric quantification of cell viability *via* annexin V/propidium iodide staining. Flow cytometry analysis showed that the combinatorial regimen with inhibitors at an initial dose of 100 μM resulted in higher viability after radiation exposure at 2 Gy than without inhibitors, as evidenced by the higher percentage of annexin V/PI negative cells in the combinatorial regimen groups (Fig. 5). The most pronounced effect was observed in MOLT-4 cells pretreated with 4 and 6. Compared to 2 Gy irradiation alone (100%), the results demonstrated that preincubation with 4 and 6 increased viability rates to 143% and 135%, respectively. As pretreatment with the tested inhibitors at 100 μM resulted in increased viability of MOLT-4 cells compared to exposure to 2 Gy IR alone, we opted to investigate the effects of a higher inhibitor dose of 200 μM. Compared with cells exposed to 2 Gy of radiation alone (considered as 100%), the percentage of viable cells in the group pretreated with inhibitors 4 and 6 at 200 μM reached 145% and 137%, respectively (Fig. 6). Furthermore, viability data showed that preincubation with amifostine at 100 μM increased MOLT-4 cell viability to 106% compared to cells exposed to 2 Gy irradiation alone. By contrast, the active moiety of amifostine, WR1065, did not exhibit distinct radioprotective effects against irradiation by 2 Gy in MOLT-4 cells at either of the concentrations of 100 μM or 200 μM. The above results obtained from the flow cytometry viability analysis suggest that WR1065, when applied alone, demonstrated significant inhibitory activity on MOLT-4 cells, resulting in a lack of any possible radioprotective effect. Collectively, these findings indicate that the synthesized piperazine derivatives (1–6) demonstrated radioprotective effects at both 100 μM and 200 μM concentrations, achieving higher protective effects than WR1065 or WR2721 administered at the same dose. This represents an advantageous characteristic for potential applications.

### Dicentric chromosome assay

**Fig. 7:**
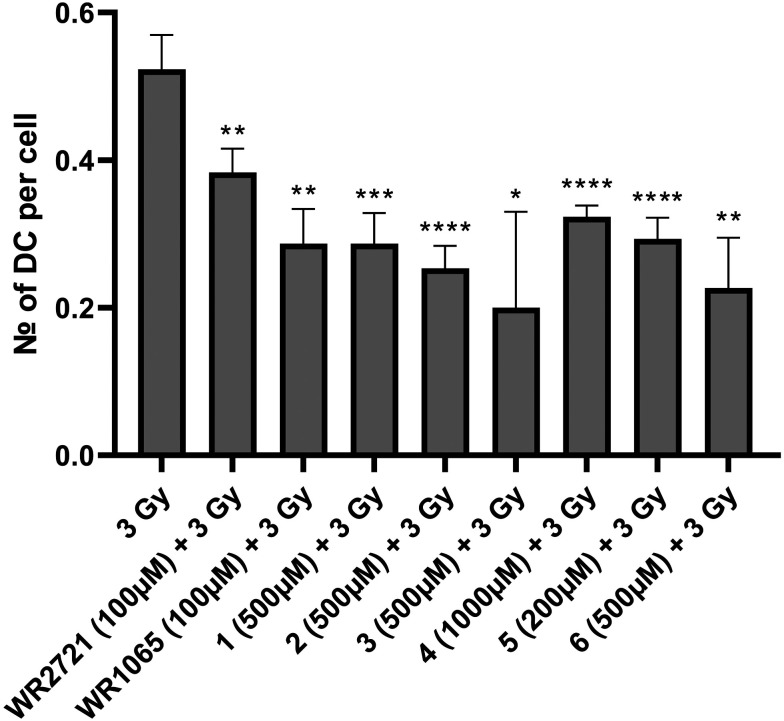
Number of dicentric chromosomes (DC) per cell after exposure to IR (3 Gy) alone or after 1 h pretreatment with the compounds at optimal non-toxic concentrations for the incubation interval of 48 h and then subjected to DCA. Error bars represent the standard deviation. The asterisks above the individual bars represent statistical significance (* *P* < 0.05, ** *P* < 0.01, *** *P* < 0.001, **** *P* < 0.0001) related to 3 Gy.

**Fig. 8:**
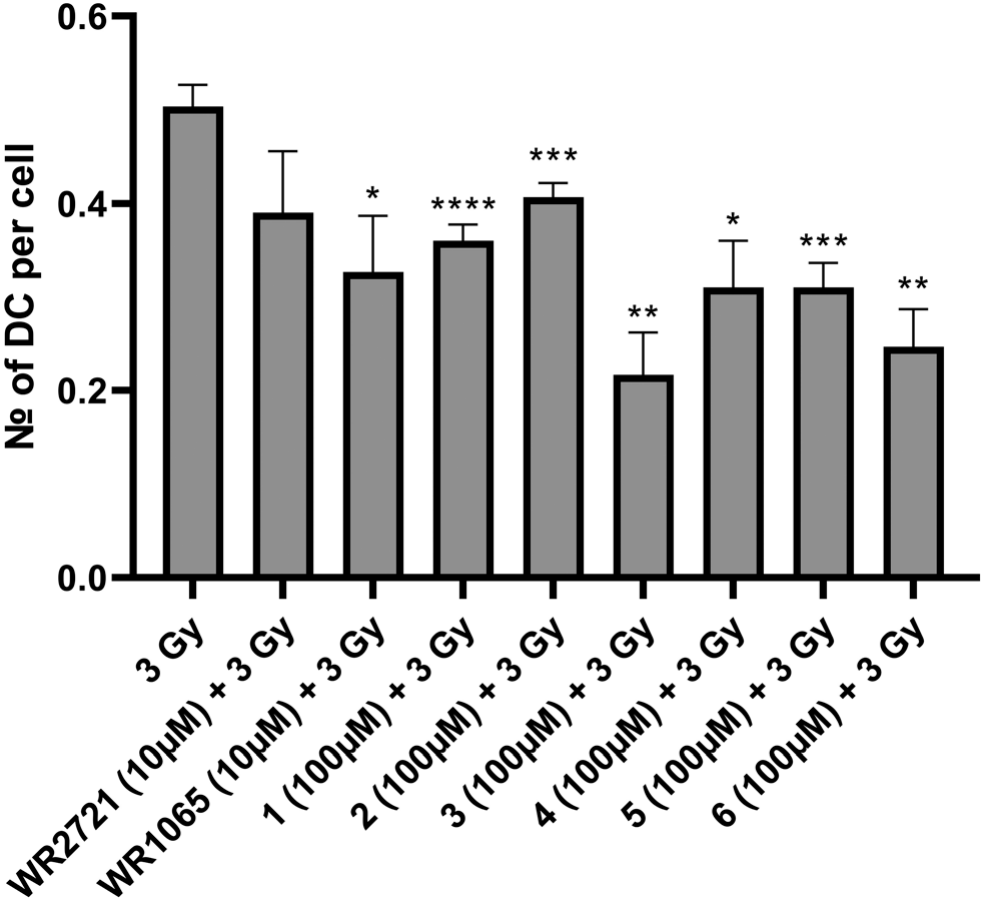
Number of dicentric chromosomes (DC) per cell after exposure to IR (3 Gy) alone or after 1 h pretreatment with the compounds at a safe concentration (100 μM for novel compounds and 10 μM for amifostine) for the incubation interval of 48 h and then subjected to DCA. Error bars represent the standard deviation. The asterisks above the individual bars represent statistical significance (* *P* < 0.05, ** *P* < 0.01, *** *P* < 0.001, **** *P* < 0.0001) related to 3 Gy.

The radioprotective effect of eight compounds was assessed using the cytogenetic method of DCA. DCA is known for its high sensitivity to radiation exposure and its specificity, and is thus considered a gold standard for biological dosimetry. It is also applicable to reveal the radioprotective potential of substances, as their effect is to protect DNA from forming dicentric chromosomes after IR exposure. This assay included six newly synthesized substances as well as amifostine (WR2721) and its active form (WR1065). WR2721 and WR1065 served as benchmarks, allowing us to measure the radioprotective effect of our compounds against these well-established radioprotectors. Whole blood from healthy human donors was utilized for DCA, and it was irradiated with 3 Gy gamma radiation after exposure to the tested compounds. Each experiment included irradiated and non-irradiated controls. The experiments were conducted at two different concentrations of the substances. For the newly synthesized compounds, 100 μM and an optimal non-toxic concentration which varied between 200–1000 μM were used. As WR2721 and WR1065 showed significantly higher toxicity, concentrations of 10 μM and 100 μM were selected for them. The results were interpreted as the number of dicentric chromosomes per cell. All novel substances exhibited a significant radioprotective effect at both concentrations, with a higher effect observed for the higher concentrations (Fig. 7 and 8).

To express the radioprotective effect the radioprotection factor (RF) was calculated as the number of DC per cell in the test sample divided by the number of DC per cell in the irradiated control (Table 2). RF expresses how many-fold the DC count in the sample decreased compared to the irradiated control. All newly synthesized compounds demonstrated a higher RF than amifostine, except for compound 2 at a concentration of 100 μM. In the first experiment with lower concentrations the active amifostine metabolite WR1065 showed higher RF than the substances 1 and 2. In the second experiment with the higher concentrations it showed higher RF than the substance 5. In all other cases the novel compounds performed better and surpassed the active metabolite WR1065. The highest RF for higher concentrations was observed for compound 3 (4.16), followed in decreasing order by 6 (2.69), 1 and 2 (both 1.97), WR1065 (1.96), 5 (1.91), 4 (1.53), and finally amifostine (1.45). Similarly, the highest RF for lower concentrations was also observed for compound 3 (2.43), followed by 6 (2.1), 4 and 5 (both 1.65), WR1065 (1.59), 1 (1.42), amifostine (1.33) and 2 (1.23).

**Table 2:** RF expressed as the number of DC per cell in the test sample divided by the number of DC per cell in the irradiated control

Compound	μM	RF	μM	RF
WR2721	10	1.33	100	1.45
WR1065	10	1.59	100	1.96
1	100	1.42	500	1.97
2	100	1.23	500	1.97
3	100	2.43	500	4.16
4	100	1.65	1000	1.53
5	100	1.65	200	1.91
6	100	2.1	500	2.69

While amifostine exhibits notable drawbacks, the advancement of radioprotective agents remains a dynamic field of research. The presented 1-(2-hydroxyethyl)piperazine derivatives hold the potential to address some of the limitations associated with amifostine. These novel substances may offer improved safety profiles and possibly higher radioprotective potential. The ongoing efforts in developing these compounds represent a hopeful avenue toward enhancing the efficacy and safety of radioprotective interventions, thereby contributing to the establishment of more effective strategies for mitigating the impact of radiation exposure in diverse scenarios.

## Conclusion

In conclusion, our study presents a significant advancement in developing novel piperazine derivatives as potent radioprotective agents. We have identified compounds with enhanced radioprotective efficacy and reduced toxicity compared to the currently employed radioprotector, amifostine. The optimization of the 1-(2-hydroxyethyl)piperazine derivatives has led to the identification of compounds that not only demonstrate superior safety profiles *in vitro* but also exhibit pronounced radioprotective effects in hematopoietic models.

The most notable findings from our study include the identification of compound 6 as a leading candidate due to its optimal balance between lipophilicity, safety, and radioprotective efficacy. Additionally, compound 3 demonstrated significant potential, especially in reducing dicentric chromosomes, indicating its strong radioprotective properties against DNA damage. Our results further highlight the importance of structural modifications, such as halogen substitution, in achieving the desired pharmacological properties. The comprehensive cytotoxicity and radioprotective assessments across a broad panel of cell lines underscore the potential of these compounds to mitigate radiation-induced damage in a diverse range of tissue types. The dual focus on compounds 6 and 3 in our study illustrates the complex landscape of developing effective radioprotective agents and promising avenues for future research.

The exploration of the radioprotective mechanism of these derivatives, particularly in relation to PUMA-dependent apoptosis pathways, opens new avenues for research not only in radiation protection but also in the treatment of diseases characterized by aberrant apoptosis. The compounds presented in this study therefore not only contribute to the field of radioprotection but may also offer insights into broader biological processes affected by IR.

Future studies will focus on further optimization of these compounds, detailed mechanistic studies, and the evaluation of their efficacy in preclinical models of ARS. Additionally, the exploration of combination therapies, leveraging the synergistic effects between these compounds and other radioprotective agents, may offer further improvements in safety and efficacy.

In summary, the novel piperazine derivatives described in this study represent promising candidates for development as radioprotective agents. Their advancement through further preclinical and clinical evaluation is eagerly anticipated, with the hope of providing enhanced protection against the harmful effects of IR, thereby benefiting individuals exposed to radiation in medical, industrial, and potential radiation emergency scenarios.

## Materials and methods

### Novel agents

#### Analysis

Reagents were purchased from Fluka Chemicals (Seelze, Germany), and Merck (Darmstadt, Germany). The solvents were acquired from penta Chemicals (Prague, Czech Republic). Thin-layer chromatography (TLC) was used to monitor the reactions, using precoated silica gel 60 F254 TLC alumina sheets. Column chromatography was performed using silica gel 0.063–0.200 mm. Compounds were identified by NMR and by HRMS. NMR spectra were determined using a Varian VNMRS500 (500 MHz for ^1^H and 125 MHz for ^13^C; Varian Co., Palo Alto, CA, USA). The chemical shifts (*δ*) are given in ppm. Coupling constants (*J*) are given in Hz. Splitting patterns are marked as s, singlet; d, doublet; t, triplet; and m, for multiplet. Mass spectra and UV purity were measured using high-performance liquid chromatography (HPLC) in tandem with mass spectrometry (MS). The system used in this study was a Dionex Ultimate 3000 RS HPLC: RS pump, RS column compartment, RS autosampler, RS diode array detector, controlled by Chromeleon (version 7.2.9 build 11 323) software (Thermo Fisher Scientific, Waltham, MA, USA) coupled with Q Exactive Plus Orbitrap mass spectrometer with Thermo Xcalibur (version 3.1.66.10.) software (Thermo Fisher Scientific, Waltham, MA, USA). Heated electrospray was used as an ionizing source with settings as follows: spray voltage 3.5 kV; capillary temperature: 262 °C; sheath gas: 55 arbitrary units; auxiliary gas: 15 arbitrary units; spare gas: 3 arbitrary units; probe heater temperature: 250 °C; max spray current: 100 mA; S-lens RF level: 50. A gradient method with a C18 column (Kinetex EVO C18, 2.1 × 50, 1.7 μm, Phenomenex, Torrance, CA, USA) was used in this study. Mobile phase A was ultrapure water of ASTM I type (resistivity 18.2 MΩ cm at 25 °C) created by Barnstead Smart2Pure 3 UV/UF apparatus (Thermo Fisher Scientific, Waltham, MA, USA) with 0.1% (v/v) formic acid (HPLC-MS grade, VWR, Radnor, PA, USA); mobile phase B was acetonitrile (HPLC-MS grade, VWR, Radnor, PA, USA) with 0.1% (v/v) of formic acid. The flow was constant at 0.4 mL min^−1^. The method begins with 0.3 min of isocratic flow of 5% B, gradually rises to 100% B in 3 min, and then remains constant at 100% B for 0.7 min. The ratio then goes back to 5% B and equilibrates for 3.5 min. The total runtime of this method is 7.5 min. The column was heated to 27 °C. Samples were dissolved in methanol (LC-MS grade, VWR, Radnor, PA, USA) at a concentration of 1 mg mL^−1^, and the sample injection was 1 μL. Purity was determined from HPLC-UV chromatogram measured at wavelength 254 nm. High-resolution mass spectra were determined by total ion current spectra from the mass spectrometer collected at 140 000 resolution in the range 105–1000 *m*/*z* in positive mode. Calculated *m*/*z* was obtained through ChemDraw Prime 19.1.1.21. MarvinSketch was used for drawing chemical structures.

#### Synthesis

The general synthetic approach consisted of a two-step procedure. In the first step, the selected substituted aryl alcohols (1 eq.) reacted with an excess of epibromohydrin (5 eq.) in the presence of a catalytic amount of piperidine. The solvent-free reaction was stirred at 135 °C for 2 h, in accordance with the procedure published by Kreighbaum *et al.*^29^ Purification of the reaction mixture was performed using column chromatography on silica, eluting with Hep : EtOAc (3 : 1) with yields ranging from 32% up to 74%, and the identity of the product was confirmed by HRMS analysis only. The purity of the intermediates was above 80%, but adequate for the following step.

In the second step, 1-(2-hydroxyethyl)piperazine (1.5 eq.) was dissolved in anhydrous acetonitrile with K_2_CO_3_ (3 eq.) and the appropriate intermediate. The reaction mixture was stirred at 85 °C for 4 h. The solid inorganic salt was removed by filtration and the organic phase concentrated under reduced pressure. The concentrated mixture was purified *via* silica column chromatography, eluting with EtOAc : MeOH : NH_3_ (25% aqueous) (6 : 2 : 0.2) to obtain oils in 47–92% yields of the second step. All of the analogs were characterized by ^1^H and ^13^C NMR and HRMS. All analyses confirmed the identity and appropriate purity (>95%) of the prepared compounds.

Finally, the acquired oils were converted to their more soluble hydrogen chloride salts by treatment with an appropriate amount (2 eq.) of 35% hydrochloric acid in methanol. The product was obtained by repeated evaporation and was dried under reduced pressure.

##### 1-(4-(2-Hydroxyethyl)piperazin-1-yl)-3-(2-methoxy-4-nitrophenoxy)propan-2-ol (1)

Brown oil (1.06 g, 82%); ^1^H NMR (500 MHz, deuterium oxide) *δ* 7.81 (td, *J* = 8.0, 7.1, 2.6 Hz, 1H), 7.70 (d, *J* = 2.6 Hz, 1H), 6.99 (d, *J* = 9.0 Hz, 1H), 4.52 (dq, *J* = 9.1, 4.6 Hz, 1H), 4.21–4.11 (m, 2H), 3.94–3.87 (m, 2H), 3.85 (s, 2H), 3.75 (s, 8H), 3.61–3.47 (m, 2H), 3.42 (dd, *J* = 6.2, 4.2 Hz, 2H); ^13^C NMR (126 MHz, D_2_O) *δ* 153.02, 148.07, 141.37, 118.35, 111.58, 107.12, 70.32, 63.73, 58.24, 56.11, 54.84, 49.04, 48.59; HRMS: *m*/*z* 356.1801 [M + H]^+^ (calculated *m*/*z* 356.1816 for [C_16_H_26_N_3_O_6_^+^]).

##### 1-(4-(2-Hydroxyethyl)piperazin-1-yl)-3-(4-methoxy-2-nitrophenoxy)propan-2-ol (2)

Brown oil (0.600 g, 77%); ^1^H NMR (500 MHz, deuterium oxide) *δ* 7.45 (d, *J* = 3.0 Hz, 1H), 7.20 (dd, *J* = 9.2, 3.1 Hz, 1H), 7.15 (d, *J* = 9.3 Hz, 1H), 4.48 (dq, *J* = 8.7, 4.4 Hz, 1H), 4.22–4.12 (m, 2H), 3.94–3.87 (m, 2H), 3.80–3.73 (m, 8H), 3.59–3.48 (m, 2H), 3.45–3.38 (m, 2H); ^13^C NMR (126 MHz, D_2_O) *δ* 152.98, 146.02, 138.87, 121.98, 117.35, 110.47, 71.56, 63.80, 58.57, 56.16, 54.84, 49.10, 48.57; HRMS: *m*/*z* 356.1808 [M + H]^+^ (calculated *m*/*z* 356.1816 for [C_16_H_26_N_3_O_6_^+^]).

##### 1-(4-(2-Hydroxyethyl)piperazin-1-yl)-3-(2-fluoro-4-nitrophenoxy)propan-2-ol (3)

Orange oil (0.960 g, 70%); ^1^H NMR (500 MHz, methanol-*d*_4_) *δ* 8.15–8.09 (m, 1H), 8.07 (dd, *J* = 11.0, 2.7 Hz, 1H), 7.40–7.32 (m, 1H), 4.63–4.55 (m, 1H), 4.33–4.25 (m, 2H), 3.99–3.86 (m, 2H), 3.84–3.82 (m, 8H), 3.67–3.48 (m, 2H), 3.49–3.44 (m, 2H); ^13^C NMR (126 MHz, MeOD) *δ* 152.16, 150.18, 141.33, 120.72, 113.85, 111.78, 71.19, 63.89, 60.07, 58.09, 55.06, 54.99; HRMS: *m*/*z* 344.1612 [M + H]^+^ (calculated *m*/*z* 344.1616 for [C_15_H_23_FN_3_O_5_^+^]).

##### 1-(4-(2-Hydroxyethyl)piperazin-1-yl)-3-(4-fluorophenoxy)propan-2-ol (4)

Brown oil (0.686 g, 80%); ^1^H NMR (500 MHz, deuterium oxide) *δ* 7.07–6.98 (m, 2H), 6.96–6.88 (m, 2H), 4.48–4.40 (m, 1H), 4.09–3.97 (m, 2H), 3.93–3.87 (m, 2H), 3.76 (s, 8H), 3.57–3.46 (m, 2H), 3.45–3.38 (m, 2H); ^13^C NMR (126 MHz, D_2_O) *δ* 154.04, 116.09, 116.06, 116.00, 115.91, 69.99, 63.82, 58.47, 58.12, 54.81, 48.49; HRMS: *m*/*z* 299.1755 [M + H]^+^ (calculated *m*/*z* 299.1776 for [C_15_H_24_FN_2_O_3_^+^]).

##### 1-(4-(2-Hydroxyethyl)piperazin-1-yl)-3-(4-chloro-3-nitrophenoxy)propan-2-ol (5)

Orange oil (0.878 g, 76%); ^1^H NMR (500 MHz, deuterium oxide) *δ* 7.54–7.47 (m, 2H), 7.18 (dd, *J* = 9.0, 3.0 Hz, 1H), 4.53–4.45 (m, 1H), 4.16–4.05 (m, 2H), 3.93–3.86 (m, 2H), 3.77 (s, 8H), 3.57–3.49 (m, 2H), 3.45–3.38 (m, 2H); ^13^C NMR (126 MHz, D_2_O) *δ* 156.91, 147.43, 132.67, 120.83, 118.42, 111.79, 69.98, 63.61, 58.26, 54.80, 48.94, 48.48, 21.24; HRMS: *m*/*z* 360.1316 [M + H]^+^ (calculated *m*/*z* 360.1321 for [C_15_H_23_ClN_3_O_5_^+^]).

##### 1-(4-(2-Hydroxyethyl)piperazin-1-yl)-3-(3-chlorophenoxy)propan-2-ol (6)

Yellow oil (1.10 g, 92%); ^1^H NMR (500 MHz, deuterium oxide) *δ* 7.25 (t, *J* = 8.4 Hz, 1H), 7.03–6.97 (m, 2H), 6.87 (ddd, *J* = 8.4, 2.4, 1.0 Hz, 1H), 4.49–4.41 (m, 1H), 4.10–3.98 (m, 2H), 3.93–3.87 (m, 2H), 3.74 (s, 8H), 3.55–3.43 (m, 2H), 3.43–3.38 (m, 2H); ^13^C NMR (126 MHz, D_2_O) *δ* 158.65, 134.40, 130.78, 121.69, 114.99, 113.25, 69.47, 63.80, 58.42, 58.12, 54.83, 48.86, 48.58; HRMS: *m*/*z* 315.1468 [M + H]^+^ (calculated *m*/*z* 315.1470 for [C_15_H_24_ClN_2_O_3_^+^]).

##### 1-(4-(2-Hydroxyethyl)piperazin-1-yl)-3-(4-(4-fluorophenoxy)phenoxy)propan-2-ol (7)

Pink oil (0.633 g, 47%); ^1^H NMR (500 MHz, deuterium oxide) *δ* 7.07–6.65 (m, 8H), 4.22–4.14 (m, 1H), 3.94–3.84 (m, 2H), 3.84–3.80 (m, 2H), 3.26–3.23 (m, 5H), 3.12 (dd, *J* = 6.2, 4.3 Hz, 2H), 3.03 (s, 5H), 2.88–2.75 (m, 2H); ^13^C NMR (126 MHz, D_2_O) *δ* 157.41, 154.38, 153.41, 150.95, 120.10, 119.53, 116.27, 116.01, 70.64, 65.63, 58.86, 57.94, 55.46, 50.69, 48.86; HRMS: *m*/*z* 391.2020 [M + H]^+^ (calculated *m*/*z* 391.2028 for [C_21_H_28_FN_2_O_4_^+^]).

##### 1-(4-(2-Hydroxyethyl)piperazin-1-yl)-3-(2,4,5-trichlorophenoxy)propan-2-ol (8)

Pink oil (1.02 g, 89%); ^1^H NMR (500 MHz, deuterium oxide) *δ* 7.51 (s, 1H), 7.18 (s, 1H), 4.51 (dt, *J* = 9.5, 4.2 Hz, 1H), 4.10 (ddd, *J* = 34.1, 10.3, 4.4 Hz, 2H), 3.93–3.87 (m, 2H), 3.77 (s, 8H), 3.62–3.50 (m, 2H), 3.46–3.38 (m, 2H); ^13^C NMR (126 MHz, D_2_O) *δ* 152.54, 130.87, 130.63, 124.35, 121.55, 115.56, 70.88, 63.67, 58.47, 54.81, 49.01, 48.52, 21.24; HRMS: *m*/*z* 383.0694 [M + H]^+^ (calculated *m*/*z* 383.0691 for [C_15_H_22_Cl_3_N_2_O_3_^+^]). Results of HRMS analysis for compounds 1–8 are shown in Fig. S3.†

### Cytotoxicity *in vitro*

Selected human tumor and non-tumor cell lines Jurkat (acute T-cell leukemia), MOLT-4 (acute lymphoblastic leukemia), A549 (lung carcinoma), HT-29 (colorectal adenocarcinoma), PANC-1 (pancreas epithelioid carcinoma), MCF-7 (breast adenocarcinoma), SAOS-2 (osteosarcoma) and MRC-5 (normal lung fibroblasts) were purchased from either American Type Culture Collection (ATCC; VA, USA) or Sigma-Aldrich (St. Louis, MO, USA) and cultured according to the provider's culture method guidelines. All cell lines were maintained at 37 °C in a humidified 5% carbon dioxide and 95% air incubator. Cells in the maximum range of either 10 passages for the adherent cell lines (A549, HT-29, PANC-1, MCF-7, SAOS-2, and MRC-5), or 20 passages for suspension cell lines (Jurkat, MOLT-4,) and in an exponential growth phase were used for this study. Screening of the antiproliferative effect and growth percent calculation were conducted as follows: each cell line was seeded at previously established optimal density (1–50 × 10^3^ cells per well) in a 96-well plate (TPP, Trasadingen, Switzerland) and cells were allowed to settle overnight. Cells were treated for 48 h with compounds at a final concentration of 100 μM. At the end of the cultivation period, the WST-1 proliferation assay (Roche, Basel, Switzerland) was performed according to the manufacturer's protocol. The absorbance was measured at 440 nm using a Tecan Spark multimode microplate reader (Tecan, Mannedorf, Switzerland). Each value is the mean of three independent experiments and represents the percentage of proliferation (100% cell proliferation for 0.1% DMSO mock-treated control cells). The GP value was calculated for each compound tested. GP represents the mean of the percentage proliferation decrease of all cell lines treated with the same compound.

### Cytotoxicity *ex vivo*

#### PBMCs separation

Whole blood from a healthy male donor was diluted 1 : 1 with Dulbecco's phosphate buffered saline (Sigma-Aldrich, St. Louis, MO, USA). The diluted blood was slowly and carefully pipetted onto an equal volume of Histopaque®-1077 (Sigma-Aldrich, St. Louis, MO, USA). The entire process was carried out at room temperature. Subsequently, centrifugation was performed at 700 × *g* with the slowest acceleration and deceleration for 20 min at 20 °C. The resulting PBMCs ring was aspirated and centrifuged at 450 × *g* for 10 min at 20 °C with normal acceleration and deceleration. The cells were washed twice with RPMI 1640 medium with GlutaMAX (Gibco by Thermo Fisher Scientific, Waltham, MA, USA). After the second wash and centrifugation, the cells were diluted with cultivation medium RPMI 1640 with GlutaMAX supplemented with 20% inactivated fetal bovine serum (both Gibco by Thermo Fisher Scientific, Waltham, MA, USA) and 1% antibiotic solution of penicillin, 10 000 IU ml^−1^ and streptomycin, 10 mg ml^−1^ (Sigma-Aldrich, St. Louis, MO, USA). At the end, the cells were counted using Türk's solution.

#### Cytotoxicity assay

PBMCs were plated into 96-well plates at a cell density of approximately 5 × 10^4^ cells per well in a volume of 100 μl. Cells were treated with tested compounds in concentrations of 10, 20, 50, 100, 200, 500, and 1000 μM to determine the optimal non-toxic concentration for each. Cells were incubated for 48 h in a humidified atmosphere with 5% CO_2_ at 37 °C. 20 μl of MTS – CellTiter 96® AQueous One Solution Cell Proliferation Assay (Promega, Madison, WI, USA) was added to each well 4 h before spectrometric analysis. Cell viability was then determined by measuring absorbance at 490 nm using the Synergy H1 instrument by BioTek. The absorbance of the cell-free culture medium was used as a blank and subtracted from the absorbance readings obtained during the experiment. The viability of untreated control cells was set as 100%.

### Annexin V/PI flow cytometry cell viability analysis

For radioprotective studies, MOLT-4 cells were irradiated alone, or irradiated after preincubation with tested compounds at 100 μM for 2 h. In the following step, at 24 h after irradiation, MOLT-4 cell viability and cell death were determined by flow cytometry using an Alexa Fluor® 488 annexin V/Dead Cell Apoptosis kit (Life Technologies, Grand Island, NY, USA) following the manufacturer's instructions. The Alexa Fluor®488 annexin V/Dead Cell Apoptosis kit employs the property of Alexa Fluor®488 conjugated to annexin V to bind to phosphatidylserine in the presence of Ca^2+^; and the ability of propidium iodide to enter cells with damaged cell membranes and bind to DNA. For each sample, a minimum of 20 000 events were acquired using a CytoFLEX LX flow cytometer (Beckman Coulter, Miami, FL, USA). List mode data were analyzed using Kaluza Analysis 2.1 software (Beckman Coulter, Miami, FL, USA).

### Dicentric chromosome assay

#### Sample preparation

Blood samples from healthy male donors were taken in heparinized tubes, with informed consent and the approval of a local ethics committee. Blood samples (1 ml) were treated with eight different substances (WR2721, WR1065, and 1–6) in two different concentrations. Non-treated irradiated and non-treated non-irradiated controls were included. All samples were exposed to a single dose of 3 Gy of gamma radiation (at the dose rate of 0.6 Gy min^−1^, using the ^60^Co Chisobalt, Chirana, Prague, Czech Republic). Samples treated with the novel compounds underwent a 60 min pre-exposure period based on prior experience, while those treated with amifostine and WR1065 (Sigma-Aldrich, St. Louis, MO, USA) had a 30 min pre-exposure period based on literature.^30,31^ The dose of 3 Gy was selected to ensure a sufficient quantity of dicentric chromosomes in lymphocytes. Immediately after irradiation, the samples were placed in a humidified atmosphere with 5% CO_2_ at 37 °C for 30 min. Treated and irradiated blood samples were added to T-25 cultivation flasks containing 10 ml RPMI 1640 with 20% fetal bovine serum and 2% phytohaemagglutinin (all Gibco by Thermo Fisher Scientific, Waltham, MA, USA). Colcemid at a final concentration of 0.25 μg ml^−1^ (Gibco by Thermo Fisher Scientific, Waltham, MA, USA) was added to each culture tube 48 h after culture initiation. Cells were cultivated for another 2 h and then harvested.

#### Dicentric chromosome analysis

At the harvest time (total time 50 h), cell pellets obtained after centrifugation at 600 × *g* for 3 min were resuspended and treated with 10 ml of prewarmed 0.075 M solution of potassium chloride (Gibco by Thermo Fisher Scientific, Waltham, MA, USA) for 20 min. After another centrifugation, cells were treated three times with fixative solution consisting of 3 : 1 (v/v) methanol : acetic acid (penta Chemicals, Prague, Czech Republic). Fixed cells were dropped on ice-cold microscope slides, air-dried overnight, and stained with 5% Giemsa solution (P-Lab, Prague, Czech Republic) for 6 min. Prepared slides were evaluated using automated metaphase finding systems. The slides with metaphases were analyzed by Axio Imager 2 (Carl Zeiss Microscopy GmbH, Göttingen, Germany) and Metafer 4 software (MetaSystems Hard & Software GmbH, Altlußheim, Germany). At first, the images of the metaphases in lower magnification were acquired (MSearch TL mode). Following that, the images of good-quality metaphases in higher resolution were acquired (AutoCapt mode). The data obtained were analyzed manually (manual selection of good-quality metaphases and dicentric chromosome detection).

### Blood samples

All experiments were performed in accordance with the guidelines of University Hospital Hradec Kralove, and experiments were approved by the ethics committee at University Hospital Hradec Kralove (No. 202305 P05). Informed consents were obtained from human participants of this study. The study procedures were performed in accordance with the Declaration of Helsinki. The blood was collected from healthy male donors under 50 years of age, who did not regularly take any medications. The obtained blood was used for the analysis of dicentric chromosomes and the separation of PBMCs for cytotoxicity assays.

### Statistical analysis

Statistical data processing and graph plotting were performed in GraphPad Prism 8.4.3. One-way ANOVA, namely Brown–Forsythe and Welch ANOVA tests, was used to evaluate the data. An unpaired *t*-test with Welch's correction was used to compare statistically significant differences between tested and control samples. A family-wise significance and confidence level of 0.05 (95% confidence interval) was used. In all graphs used, the error bars represent the standard deviation. Asterisks shown above a graph column indicate a statistically significant difference from the control group. One asterisk corresponds to a *P* value lower than 0.05, two asterisks correspond to *P* < 0.01, three asterisks to *P* < 0.001, and four asterisks to *P* < 0.0001.

## Abbreviations

ARS: Acute radiation syndrome
DCA: Dicentric chromosome assay
DMSO: Dimethyl sulfoxide
GP: Growth percent
HPLC: High-performance liquid chromatography
HRMS: High-resolution mass spectrometry
IR: Ionizing radiation
NMR: Nuclear magnetic resonance
PBMCs: Peripheral blood mononuclear cells
RF: Radioprotection factor
TLC: Thin-layer chromatography

## Data availability

Raw data that support the findings of this study are available from corresponding author, upon reasonable request.

## Declaration of generative AI and AI-assisted technologies in the writing process

During the preparation of this manuscript, the authors used ChatGPT-4 to enhance the language quality, considering the authors are not native English speakers. After using this tool, the authors reviewed and edited the content as needed and took full responsibility for the content of the publication.

## Author contributions

Vojtěch Chmil: formal analysis, investigation, visualisation, writing – original draft. Natálie Živná: investigation, writing – original draft. Marcela Milanová: investigation, methodology, writing – original draft. Alžběta Filipová: investigation. Jaroslav Pejchal: conceptualization. Lukáš Prchal: investigation. Darina Muthná: investigation, methodology, writing – original draft. Vít Řeháček: resources. Martina Řezáčová: conceptualization, writing – review & editing. Jan Marek: conceptualization, methodology, writing – review & editing. Aleš Tichý: conceptualization, funding acquisition, project administration, supervision, writing – review & editing. Radim Havelek: investigation, methodology, writing – original draft.

## Conflicts of interest

The authors declare that they have no known competing financial interests or personal relationships that could appear to influence the work reported in this paper.

## Supplementary Material

MD-015-D4MD00311J-s001
