# Supplementary material for: Second-generation piperazine derivatives as promising radiation countermeasures
Source: RSC Med Chem. 2024 Jul 11;15(8):2855–66. doi: 10.1039/d4md00311j (PMC11324046; doi:10.1039/d4md00311j)
Supplement: MD-015-D4MD00311J-s001 [file MD-015-D4MD00311J-s001.pdf]

Electronic Supplementary Material (ESI) for RSC Medicinal Chemistry.
This journal is © The Royal Society of Chemistry 2024

# Supplementary Material

**Second-generation piperazine derivatives as promising radiation countermeasures**

**Authors:**

Vojtěch Chmil[a], Natálie Živná[b,c], Marcela Milanová[a], Alžběta Filipová[a], Jaroslav Pejchal[b], Lukáš Prchal[c], Darina Muthná[d], Vít Řeháček[e], Martina Řezáčová[d], Jan Marek[c,f], Aleš Tichý[a,c], Radim Havelek[d*]

* Corresponding author: e-mail: havelekr@lfhk.cuni.cz

**Affiliations:**

[a]*Department of Radiobiology, Military Faculty of Medicine, University of Defence in Brno, Trebesska 1575, 500 05 Hradec Kralove, Czech Republic*

[b]*Department of Toxicology and Military Pharmacy, Military Faculty of Medicine, University of Defence in Brno, Trebesska 1575, 500 05 Hradec Kralove, Czech Republic*

[c]*Biomedical Research Centre, University Hospital Hradec Kralove, Sokolska 581, 500 05 Hradec Kralove, Czech Republic*

[d]*Department of Medical Biochemistry, Faculty of Medicine in Hradec Kralove, Charles University, Simkova 870, 500 03 Hradec Kralove, Czech Republic*

[e]*Transfusion Center, University Hospital Hradec Kralove, Sokolska 581, 500 05 Hradec Kralove – Novy Hradec Kralove, Czech Republic*

[f]*Department of Epidemiology, Military Faculty of Medicine, University of Defence in Brno, Trebesska 1575, 500 05 Hradec Kralove, Czech Republic*

**Supplementary Fig. 1.** Proliferation of individual cell lines exposed to the compounds at 100 µM for 48 h. All data are an average of at least three independent measurements ± standard deviation.

| Average viability (%) | | | | | | | | | | |
|---|---|---|---|---|---|---|---|---|---|---|
| | **WR2721** | **WR1065** | **1** | **2** | **3** | **4** | **5** | **6** | **7** | **8** |
| Jurkat | 10 ± 12 | 1 ± 0 | 91 ± 2 | 98 ± 3 | 92 ± 4 | 100 ± 3 | 85 ± 3 | 89 ± 5 | 2 ± 0 | 28 ± 4 |
| MOLT-4 | 71 ± 21 | 3 ± 3 | 92 ± 8 | 90 ± 7 | 84 ± 11 | 102 ± 16 | 84 ± 8 | 89 ± 16 | 6 ± 5 | 41 ± 8 |
| A549 | 5 ± 0 | 3 ± 0 | 112 ± 8 | 111 ± 11 | 106 ± 11 | 112 ± 14 | 109 ± 9 | 99 ± 11 | 6 ± 2 | 92 ± 12 |
| HT-29 | 27 ± 3 | 20 ± 2 | 141 ± 42 | 103 ± 3 | 94 ± 9 | 99 ± 3 | 95 ± 6 | 85 ± 7 | 12 ± 2 | 33 ± 3 |
| PANC-1 | 8 ± 4 | 3 ± 1 | 108 ± 3 | 104 ± 3 | 90 ± 4 | 97 ± 1 | 100 ± 3 | 94 ± 3 | 55 ± 6 | 101 ± 2 |
| MCF-7 | 7 ± 2 | 2 ± 0 | 100 ± 9 | 97 ± 8 | 91 ± 12 | 102 ± 5 | 104 ± 11 | 86 ± 12 | 7 ± 7 | 41 ± 7 |
| SAOS-2 | 9 ± 3 | 7 ± 4 | 101 ± 16 | 103 ± 9 | 92 ± 14 | 102 ± 6 | 89 ± 3 | 120 ± 26 | 15 ± 5 | 42 ± 5 |
| MRC-5 | 8 ± 4 | 3 ± 1 | 111 ± 9 | 105 ± 1 | 103 ± 6 | 103 ± 2 | 98 ± 3 | 97 ± 1 | 0 ± 1 | 15 ± 10 |

**Supplementary Fig. 2.** Viability in % of PBMCs after 48 h exposure to tested compounds. The viability of untreated control is 100%. All data are an average of at least three independent measurements ± standard deviation.

| | Average viability (%) | | | | | | | |
|---|---|---|---|---|---|---|---|---|
| **[µM]** | **WR2721** | **WR1065** | **1** | **2** | **3** | **4** | **5** | **6** |
| **0** | 100 ± 0 | 100 ± 0 | 100 ± 0 | 100 ± 0 | 100 ± 0 | 100 ± 0 | 100 ± 0 | 100 ± 0 |
| **10** | 93 ± 17 | 57 ± 39 | 114 ± 14 | 102 ± 12 | 98 ± 5 | 104 ± 18 | 121 ± 25 | 94 ± 12 |
| **20** | 75 ± 30 | 47 ± 34 | 110 ± 11 | 100 ± 11 | 95 ± 14 | 103 ± 26 | 99 ± 8 | 96 ± 5 |
| **50** | 37 ± 9 | 19 ± 17 | 111 ± 15 | 99 ± 6 | 90 ± 9 | 112 ± 30 | 111 ± 7 | 99 ± 4 |
| **100** | 33 ± 12 | 16 ± 14 | 101 ± 17 | 98 ± 8 | 92 ± 6 | 107 ± 30 | 102 ± 9 | 99 ± 7 |
| **200** | 35 ± 17 | 21 ± 8 | 102 ± 21 | 99 ± 11 | 92 ± 5 | 110 ± 33 | 107 ± 5 | 96 ± 6 |
| **500** | 37 ± 20 | 27 ± 9 | 97 ± 22 | 93 ± 3 | 92 ± 9 | 110 ± 28 | 79 ± 3 | 94 ± 7 |
| **1000** | n/a | n/a | 60 ± 10 | 78 ± 4 | 81 ± 11 | 105 ± 10 | 44 ± 11 | 51 ± 4 |
| **2000** | n/a | n/a | n/a | n/a | n/a | 53 ± 5 | n/a | n/a |
| **5000** | n/a | n/a | n/a | n/a | n/a | 39 ± 5 | n/a | n/a |

**Supplementary Fig. 3.** HRMS analysis of compounds **1 - 8**

1-(4-(2-hydroxyethyl)piperazin-1-yl)-3-(2-methoxy-4-nitrophenoxy)propan-2-ol (**1**)

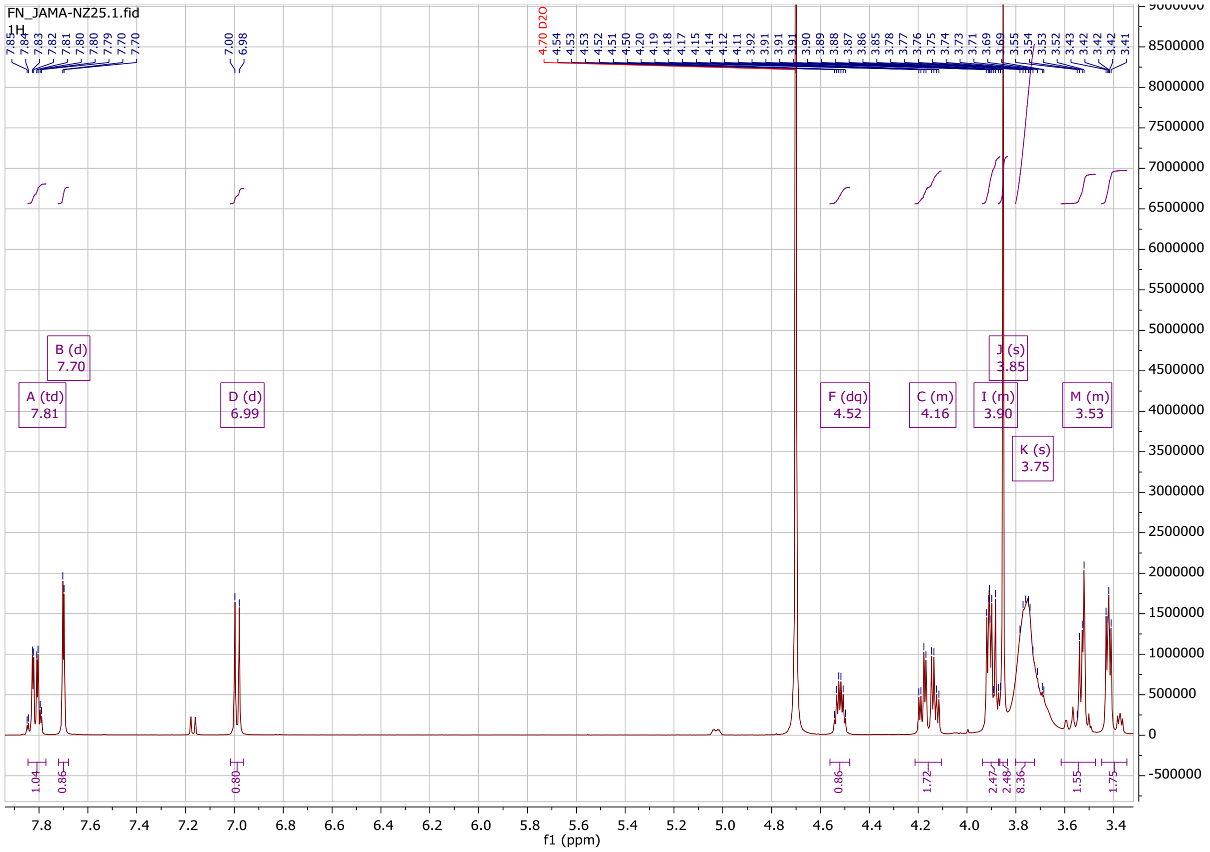

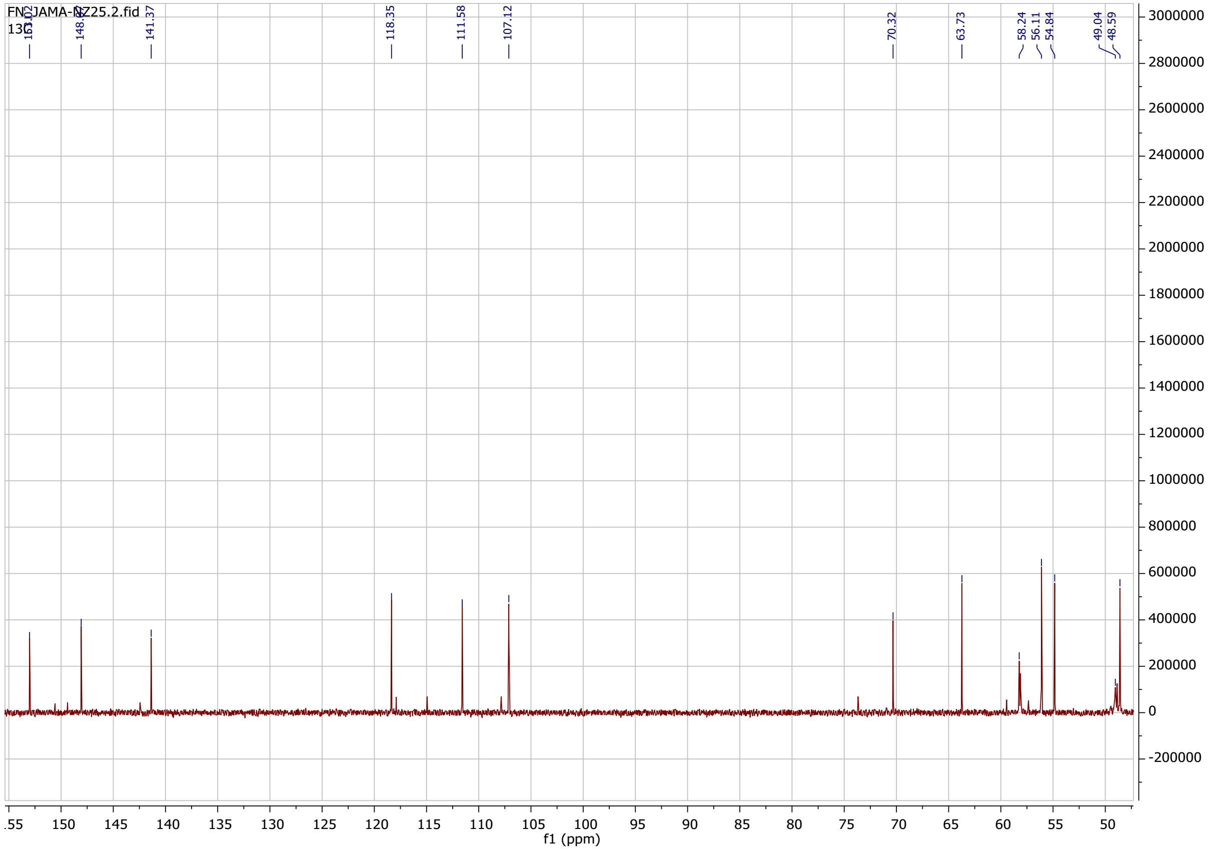
FN JAMA-NZ25.2.fid
13C
153.02
148.07
141.37
118.35
111.58
107.12
70.32
63.73
58.24
56.11
54.84
49.04
48.59
3000000
2800000
2600000
2400000
2200000
2000000
1800000
1600000
1400000
1200000
1000000
800000
600000
400000
200000
0
-200000
155
150
145
140
135
130
125
120
115
110
105
100
95
90
85
80
75
70
65
60
55
50
f1 (ppm)

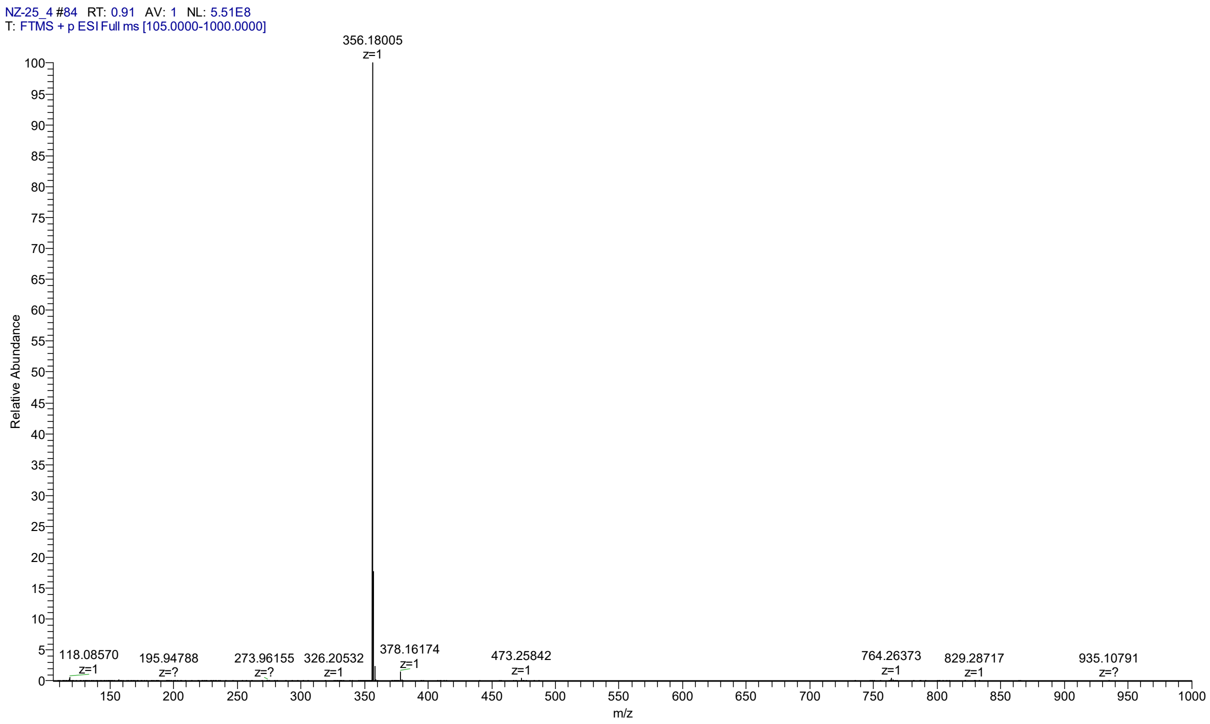
NZ-25_4 #84 RT: 0.91 AV: 1 NL: 5.51E8
T: FTMS + p ESI Full ms [105.0000-1000.0000]
356.18005
z=1
118.08570
z=1
195.94788
z=?
273.96155
z=?
326.20532
z=1
378.16174
z=1
473.25842
z=1
764.26373
z=1
829.28717
z=1
935.10791
z=?
Relative Abundance
m/z

1-(4-(2-hydroxyethyl)piperazin-1-yl)-3-(4-methoxy-2-nitrophenoxy)propan-2-ol (**2**)

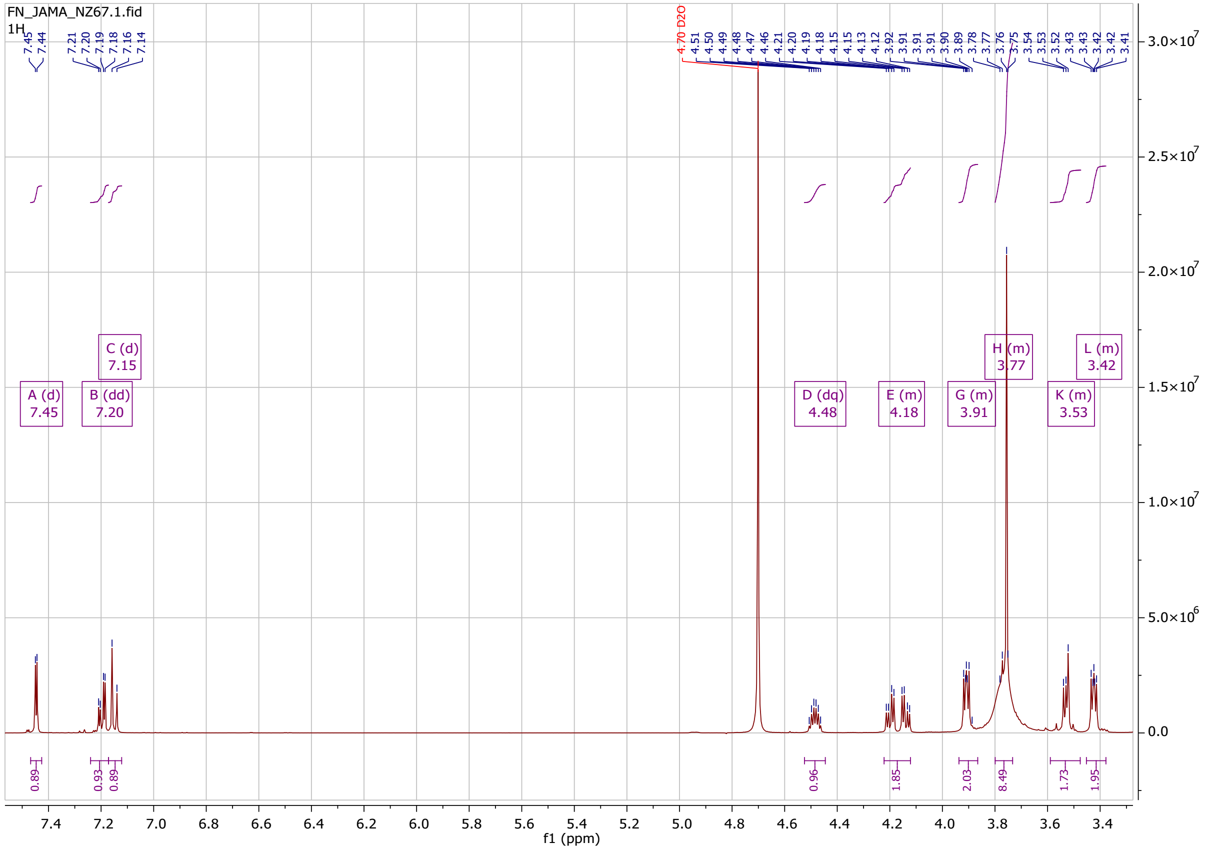

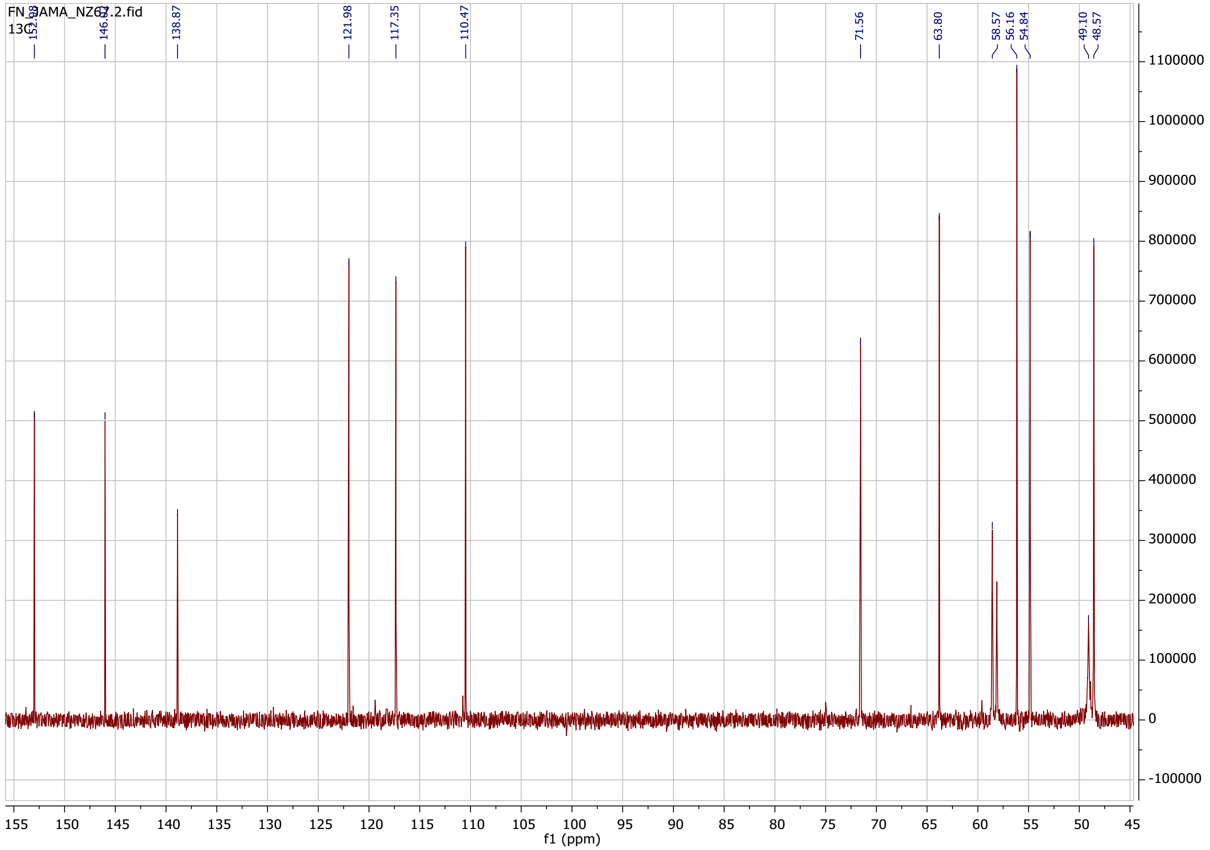
FN_JAMA_NZ67.2.fid
13C
152.98
146.07
138.87
121.98
117.35
110.47
71.56
63.80
58.57
56.16
54.84
49.10
48.57
f1 (ppm)

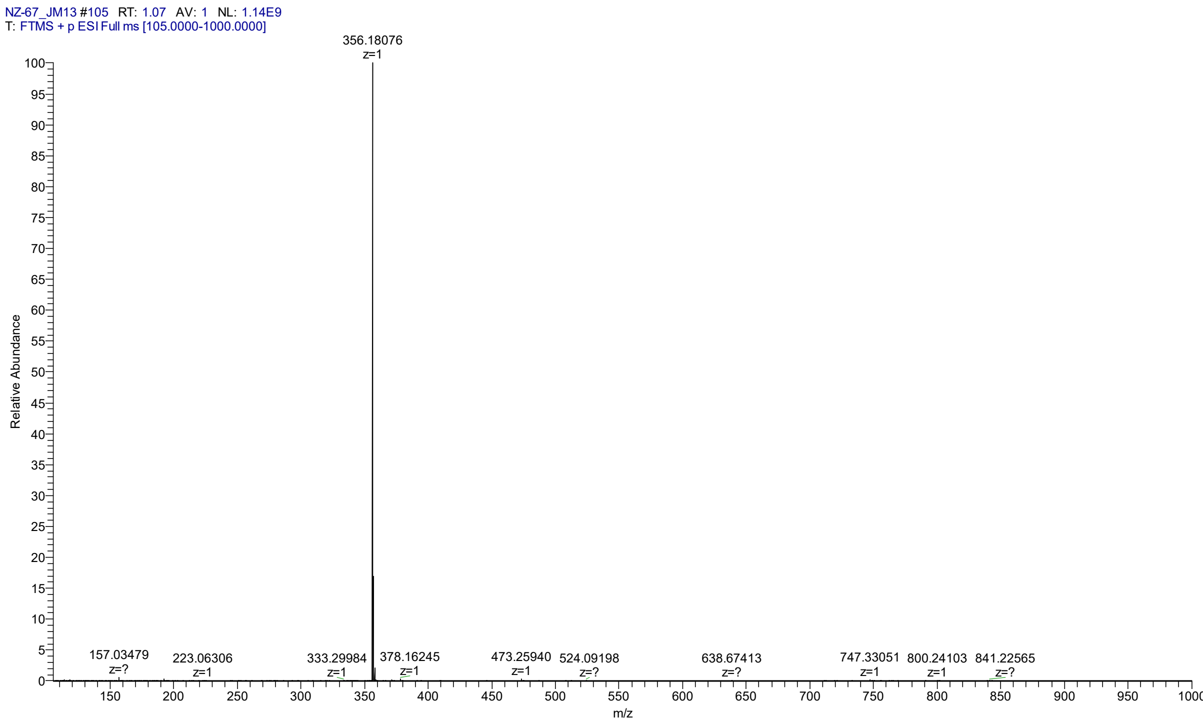

NZ-67_JM13 #105 RT: 1.07 AV: 1 NL: 1.14E9
T: FTMS + p ESI Full ms [105.0000-1000.0000]
356.18076
z=1
Relative Abundance
157.03479
z=?
223.06306
z=1
333.29984
z=1
378.16245
z=1
473.25940
z=1
524.09198
z=?
638.67413
z=?
747.33051
z=1
800.24103
z=1
841.22565
z=?
m/z

# 1-(4-(2-hydroxyethyl)piperazin-1-yl)-3-(2-fluoro-4-nitrophenoxy)propan-2-ol (**3**)

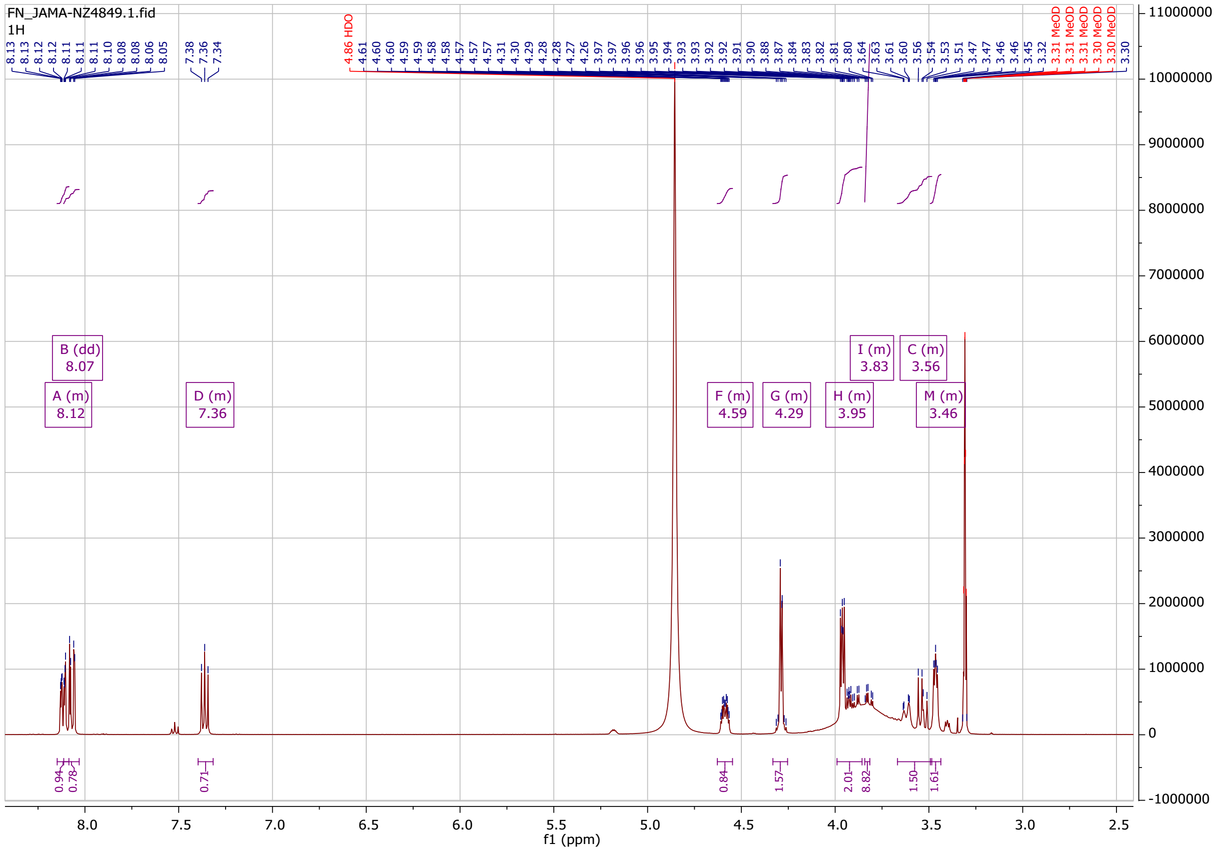

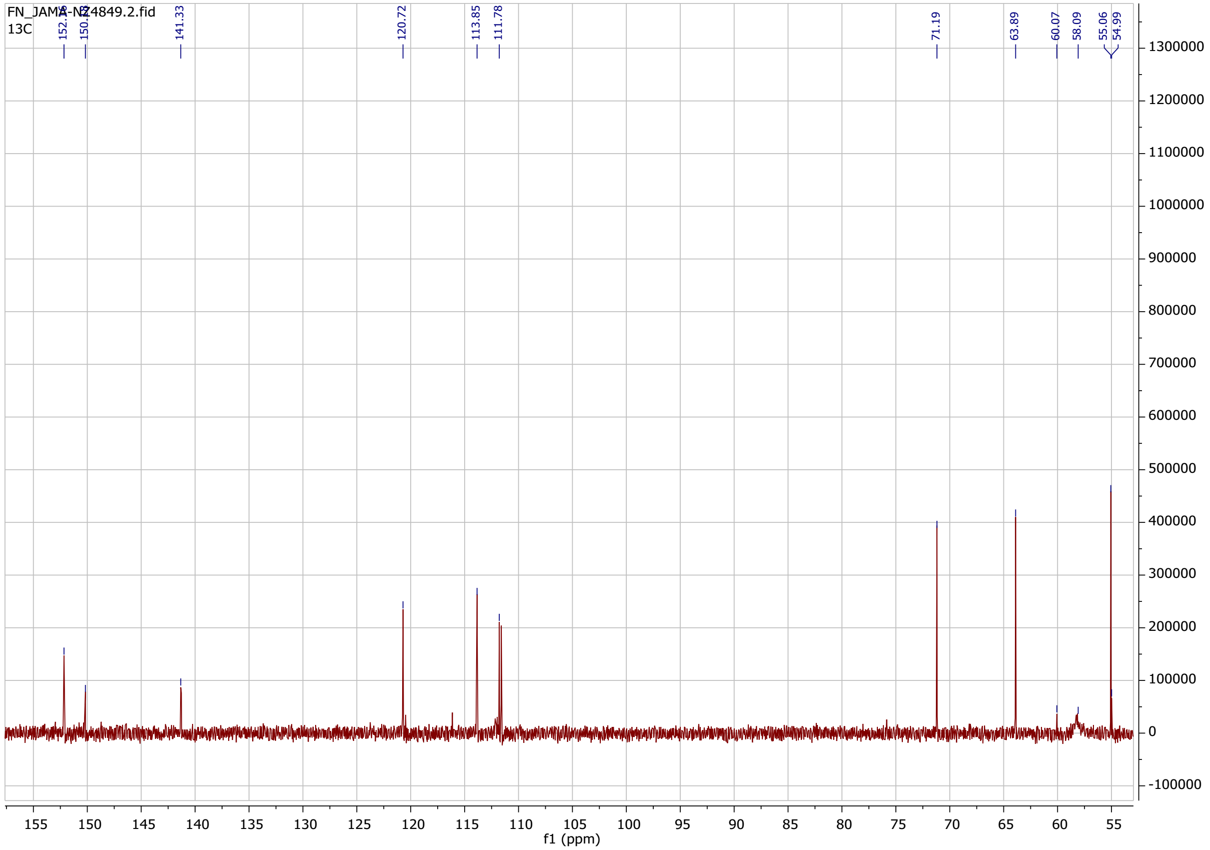
FN_JAMA-NZ4849.2.fid
13C
141.33
120.72
113.85
111.78
71.19
63.89
60.07
58.09
55.06
54.99
1300000
1200000
1100000
1000000
900000
800000
700000
600000
500000
400000
300000
200000
100000
0
-100000
155
150
145
140
135
130
125
120
115
110
105
100
95
90
85
80
75
70
65
60
55
f1 (ppm)

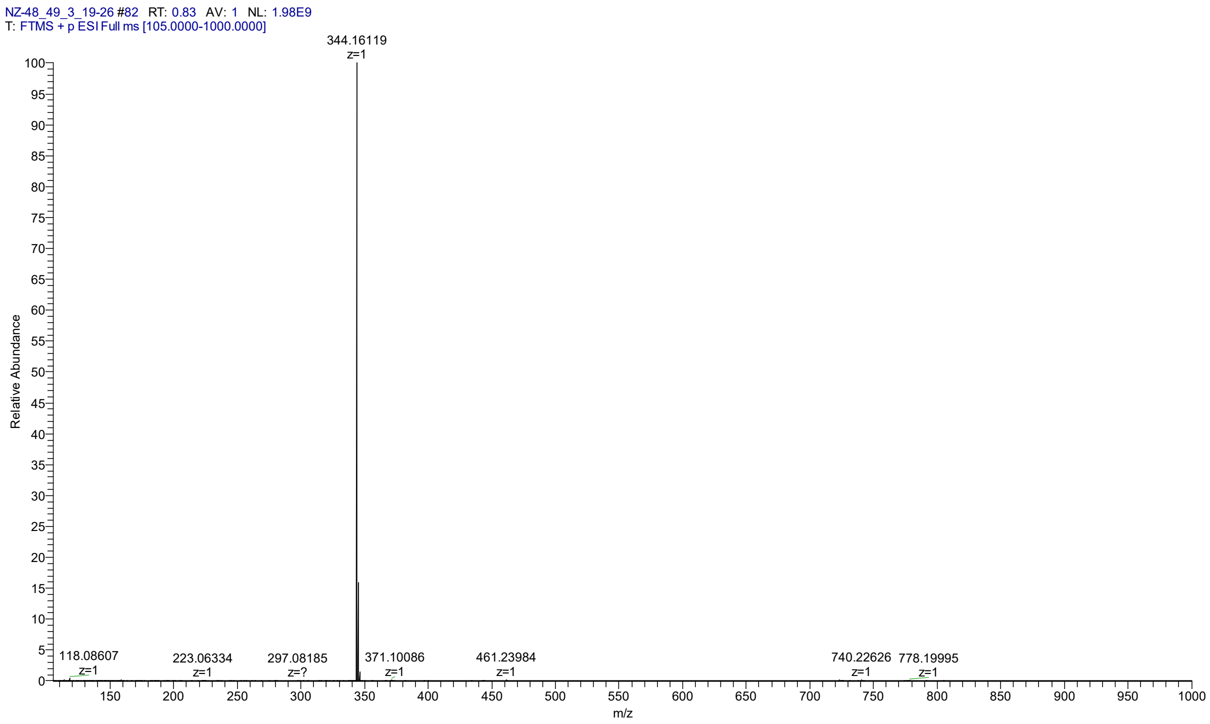

NZ-48_49_3_19-26 #82 RT: 0.83 AV: 1 NL: 1.98E9
T: FTMS + p ESI Full ms [105.0000-1000.0000]
344.16119
z=1
118.08607
z=1
223.06334
z=1
297.08185
z=?
371.10086
z=1
461.23984
z=1
740.22626
z=1
778.19995
z=1
Relative Abundance
m/z

1-(4-(2-hydroxyethyl)piperazin-1-yl)-3-(4-fluorophenoxy)propan-2-ol (**4**)

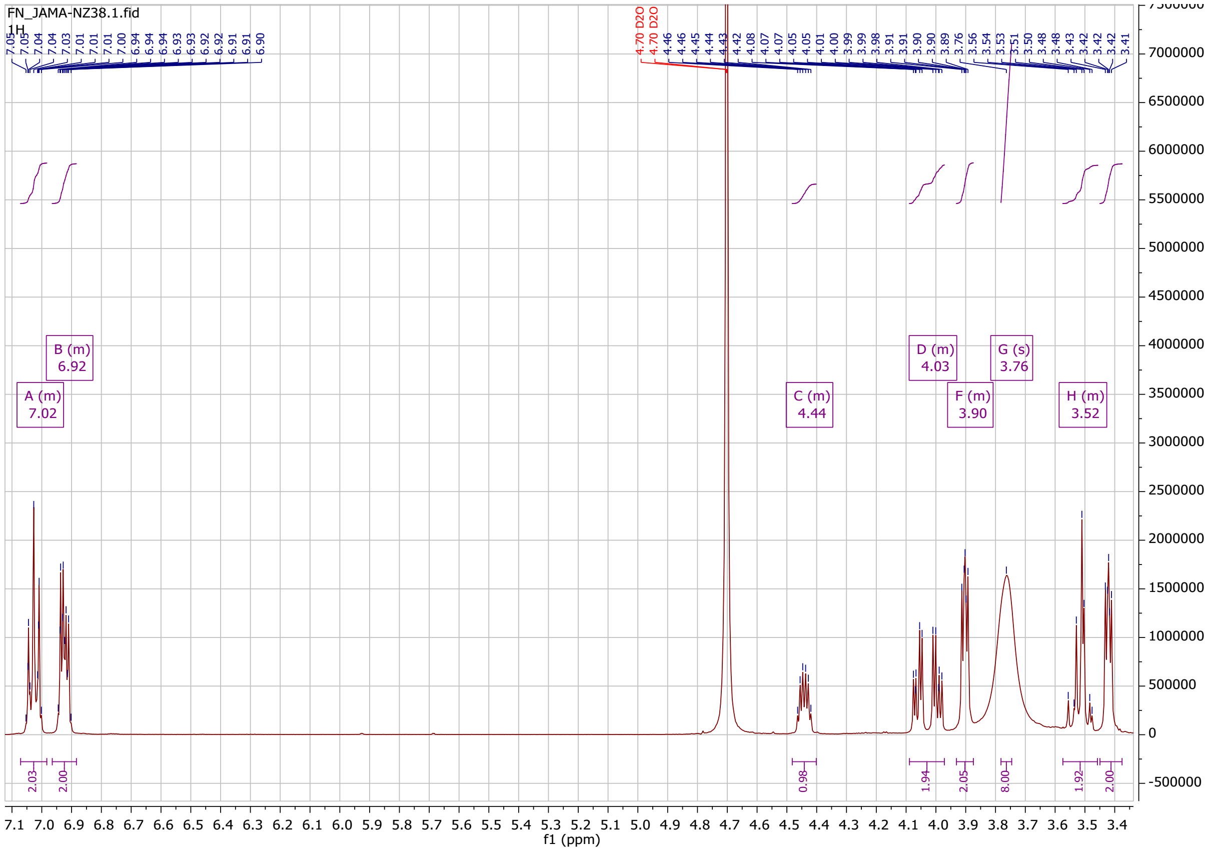

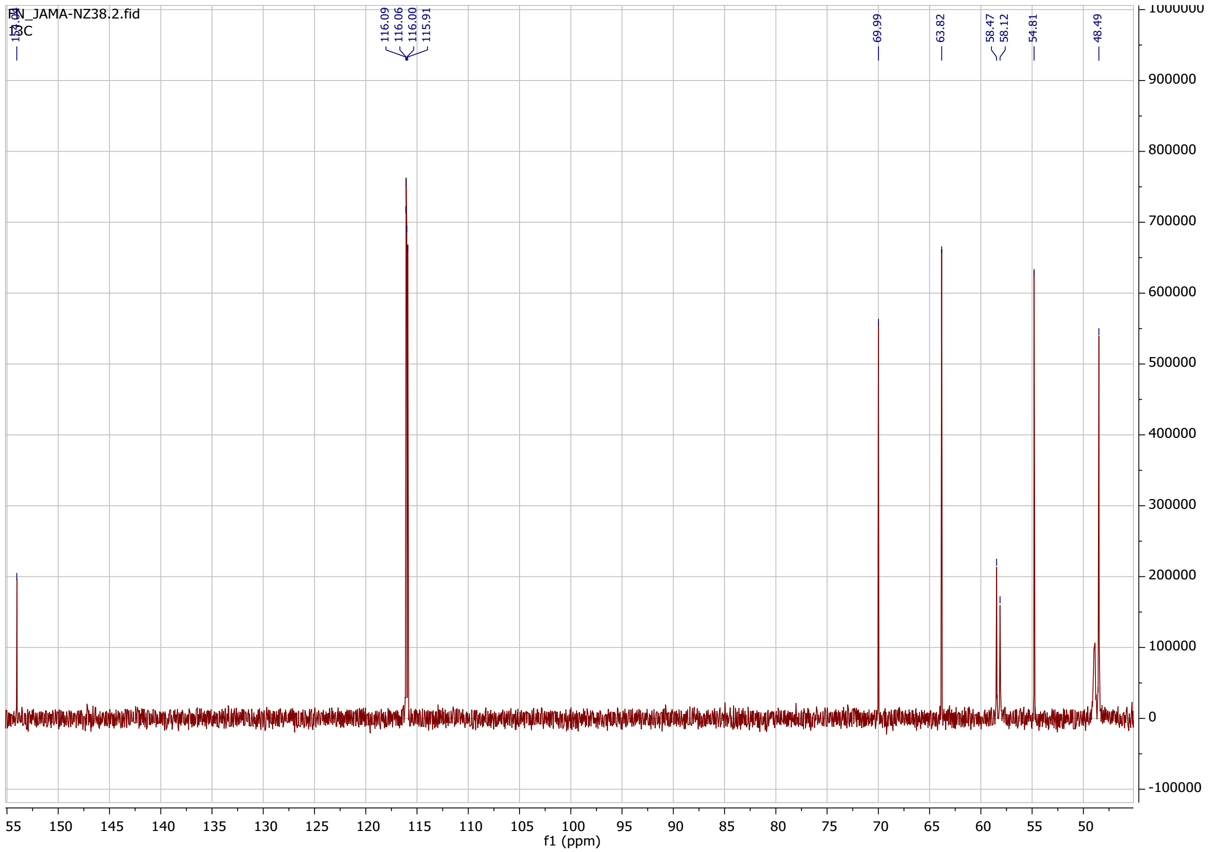

FN_JAMA-NZ38.2.fid
13C
154.04
116.09
116.06
116.00
115.91
69.99
63.82
58.47
58.12
54.81
48.49
1000000
900000
800000
700000
600000
500000
400000
300000
200000
100000
0
-100000
55
150
145
140
135
130
125
120
115
110
105
100
95
90
85
80
75
70
65
60
55
50
f1 (ppm)

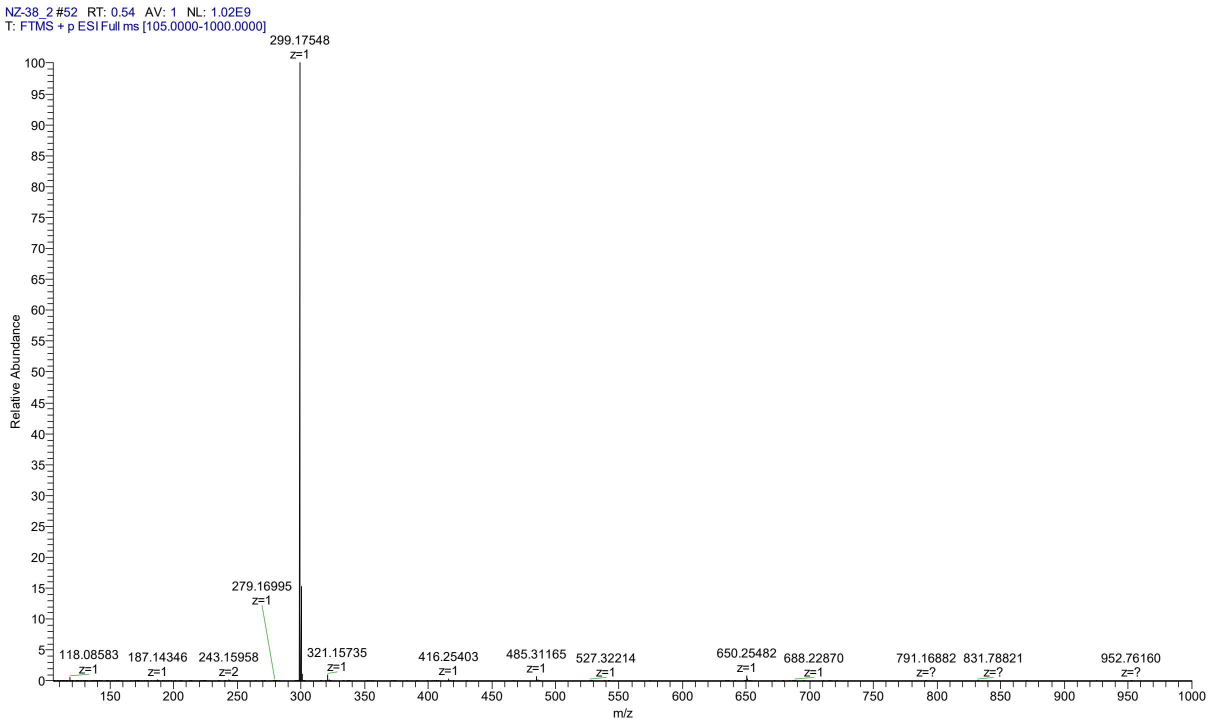

NZ-38_2 #52 RT: 0.54 AV: 1 NL: 1.02E9
T: FTMS + p ESI Full ms [105.0000-1000.0000]
299.17548
z=1
279.16995
z=1
118.08583
z=1
187.14346
z=1
243.15958
z=2
321.15735
z=1
416.25403
z=1
485.31165
z=1
527.32214
z=1
650.25482
z=1
688.22870
z=1
791.16882
z=?
831.78821
z=?
952.76160
z=?
Relative Abundance
m/z

1-(4-(2-hydroxyethyl)piperazin-1-yl)-3-(4-chloro-3-nitrophenoxy)propan-2-ol (**5**)

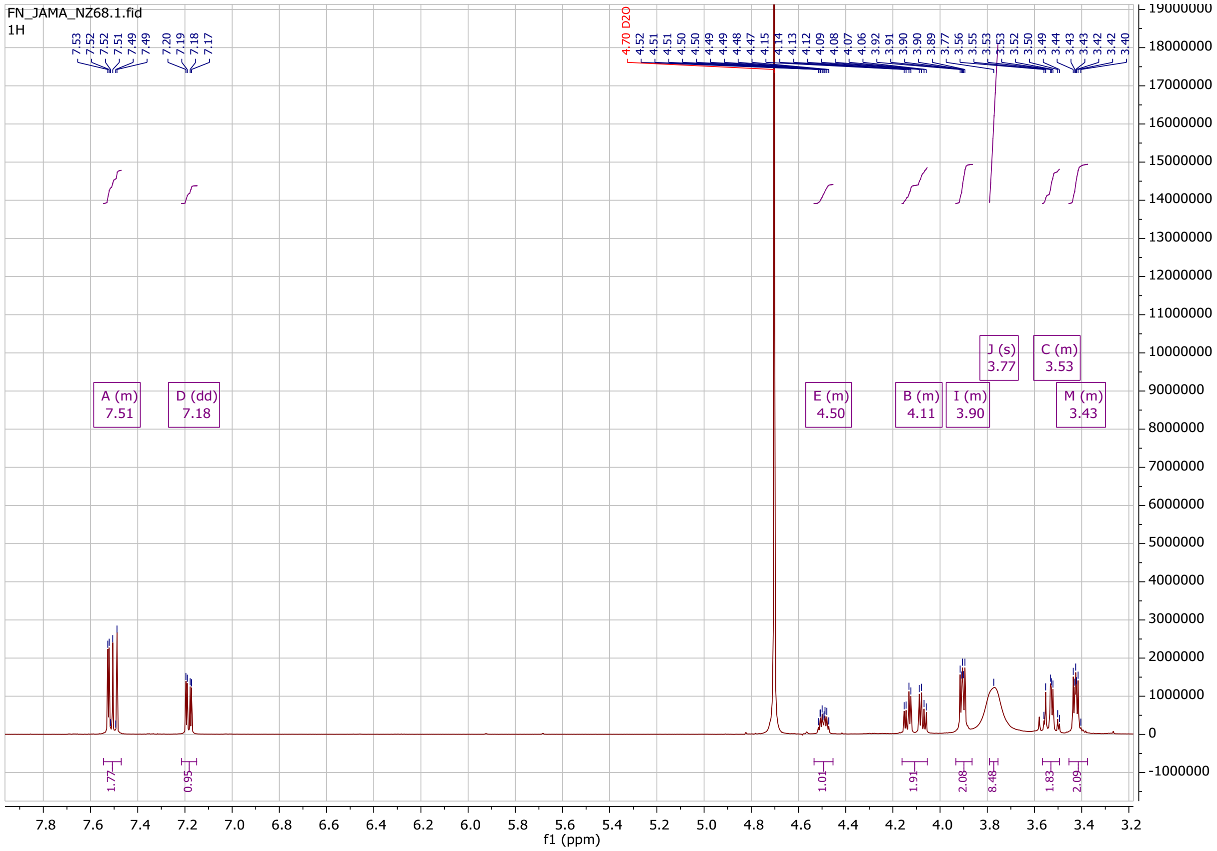

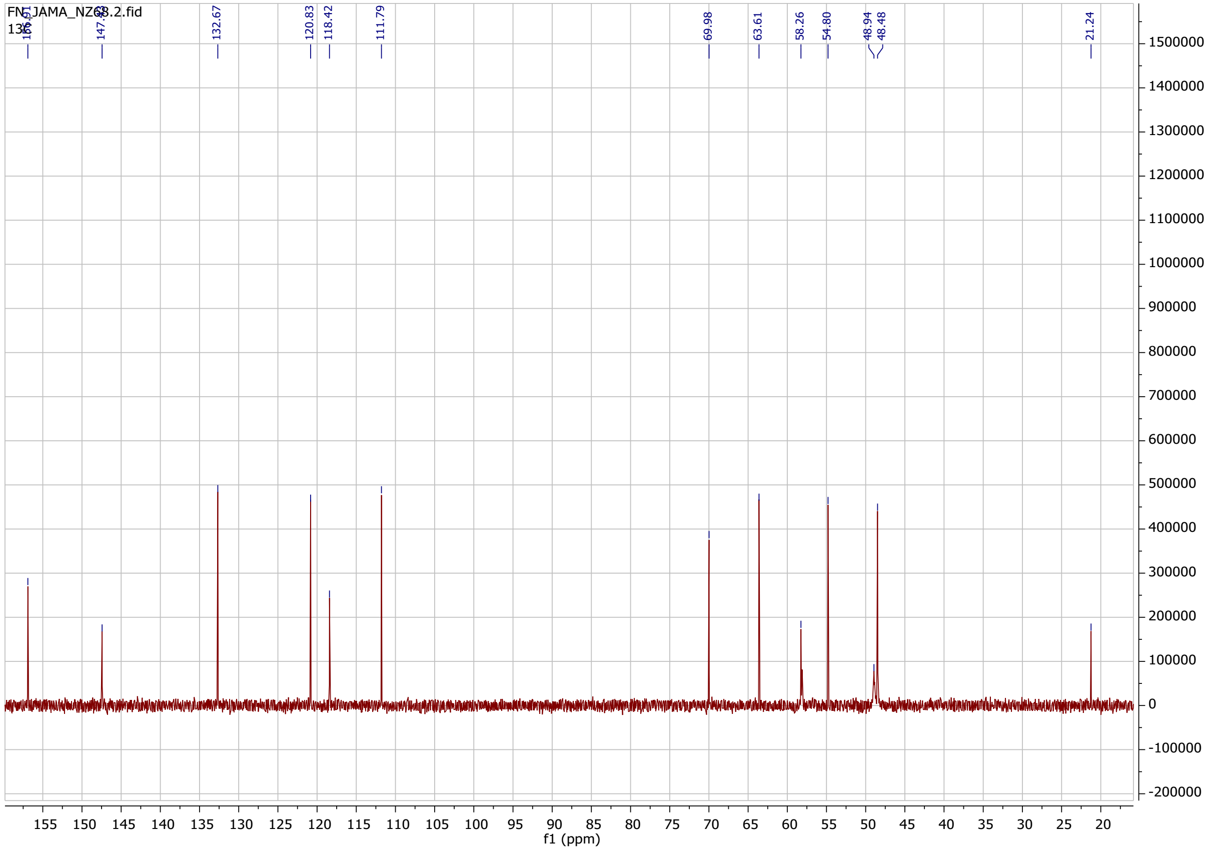
FN_JAMA_NZ68.2.fid
13C
156.91
147.43
132.67
120.83
118.42
111.79
69.98
63.61
58.26
54.80
48.94
48.48
21.24
1500000
1400000
1300000
1200000
1100000
1000000
900000
800000
700000
600000
500000
400000
300000
200000
100000
0
-100000
-200000
155 150 145 140 135 130 125 120 115 110 105 100 95 90 85 80 75 70 65 60 55 50 45 40 35 30 25 20
f1 (ppm)

NZ-68_JM14 #175 RT: 1.82 AV: 1 NL: 7.09E8
T: FTMS + p ESI Full ms [105.0000-1000.0000]

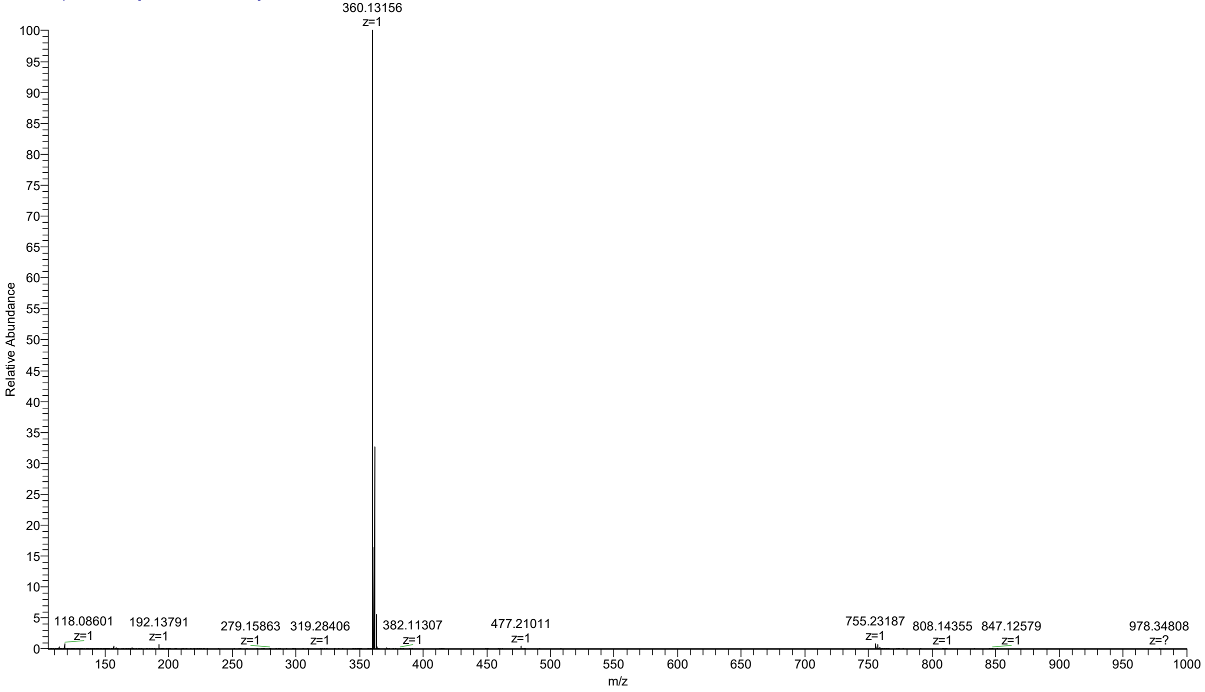

1-(4-(2-hydroxyethyl)piperazin-1-yl)-3-(3-chlorophenoxy)propan-2-ol (**6**)

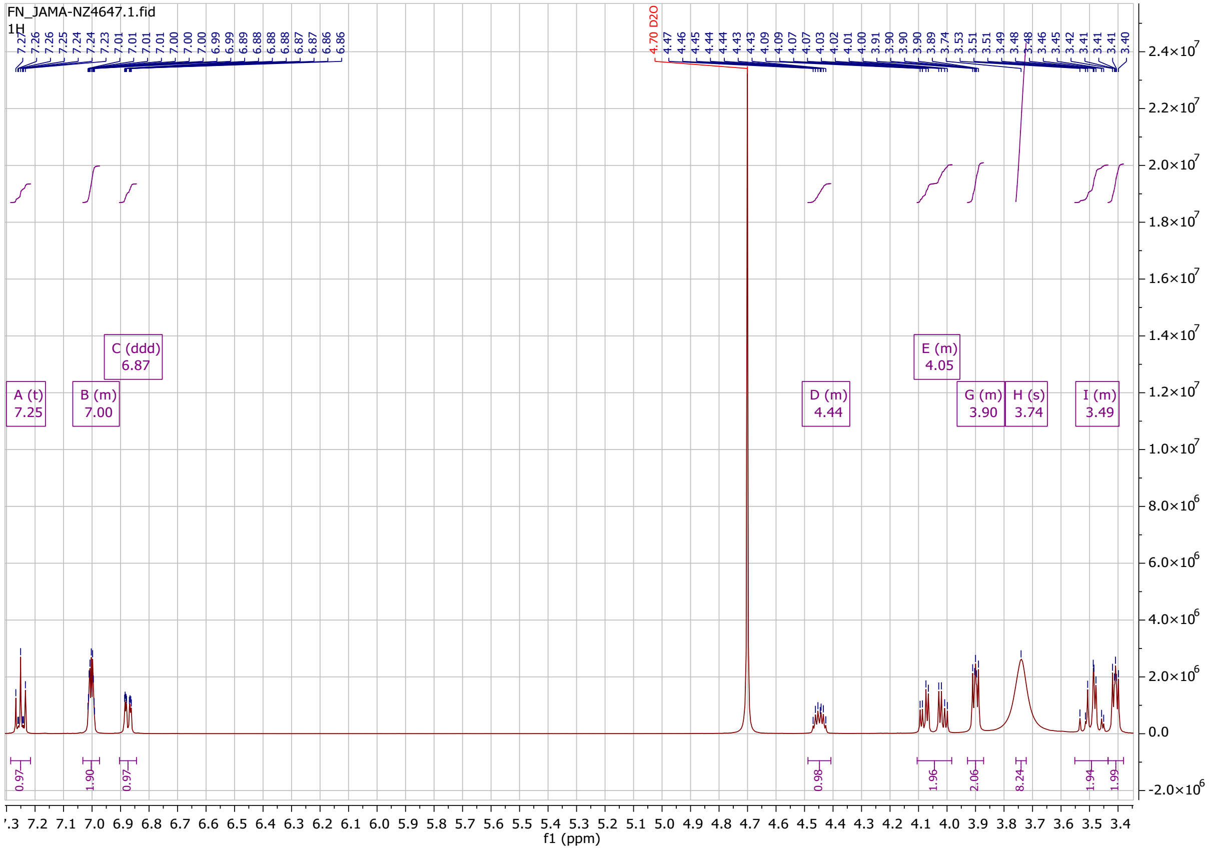

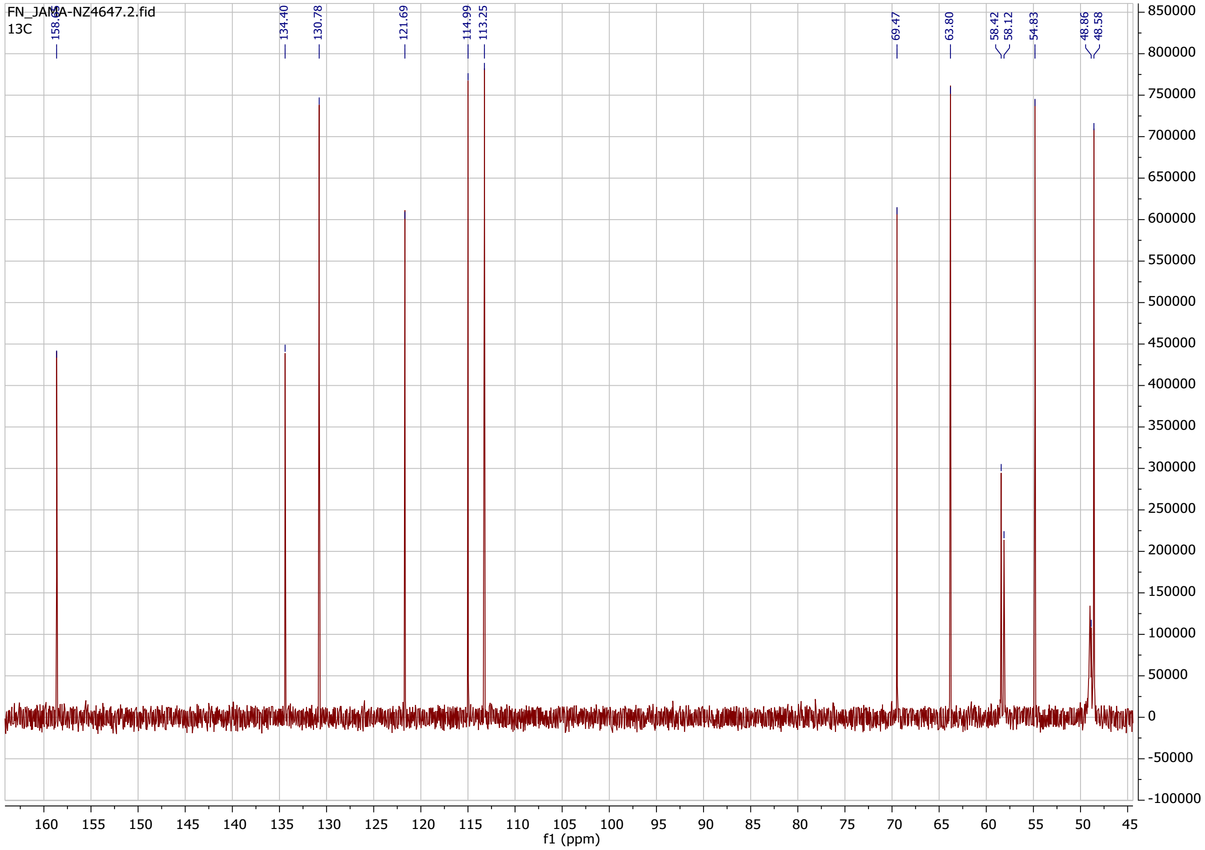
FN_JAMA-NZ4647.2.fid
13C
134.40
130.78
121.69
114.99
113.25
69.47
63.80
58.42
58.12
54.83
48.86
48.58
f1 (ppm)

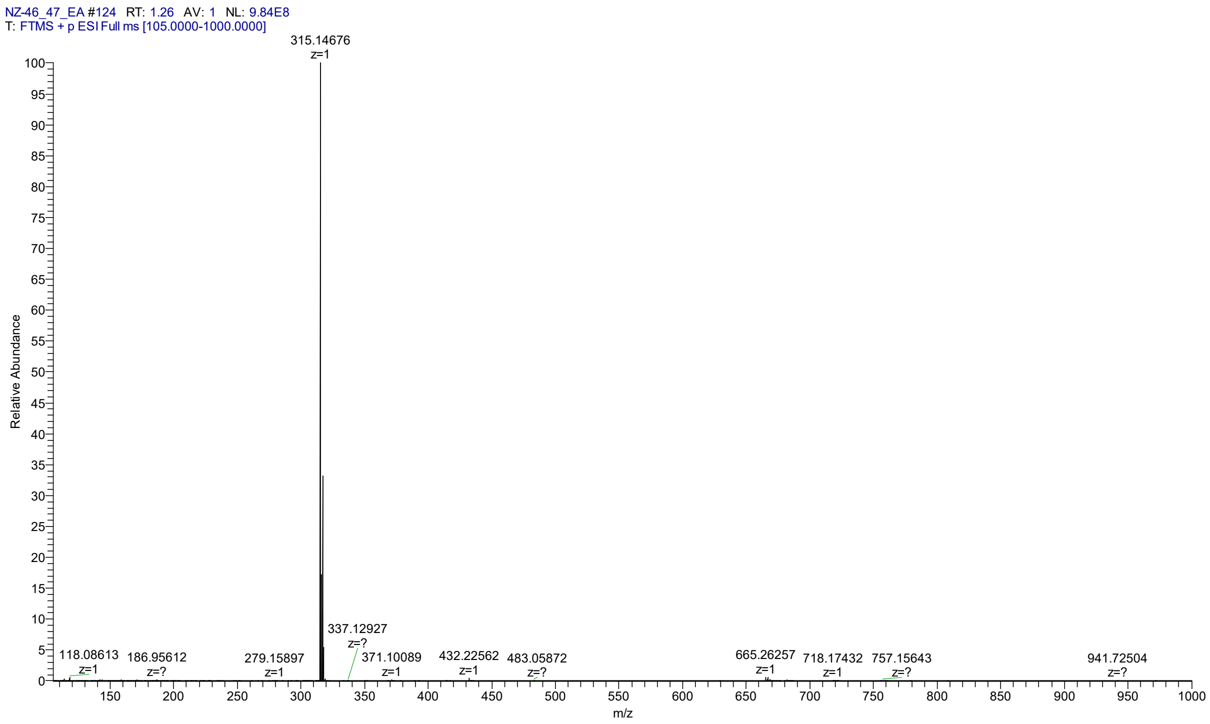
NZ-46_47_EA #124 RT: 1.26 AV: 1 NL: 9.84E8
T: FTMS + p ESI Full ms [105.0000-1000.0000]
315.14676
z=1
337.12927
z=?
118.08613
z=1
186.95612
z=?
279.15897
z=1
371.10089
z=1
432.22562
z=1
483.05872
z=?
665.26257
z=1
718.17432
z=1
757.15643
z=?
941.72504
z=?
Relative Abundance
m/z

1-(4-(2-hydroxyethyl)piperazin-1-yl)-3-(4-(4-fluorophenoxy)phenoxy)propan-2-ol (**7**)

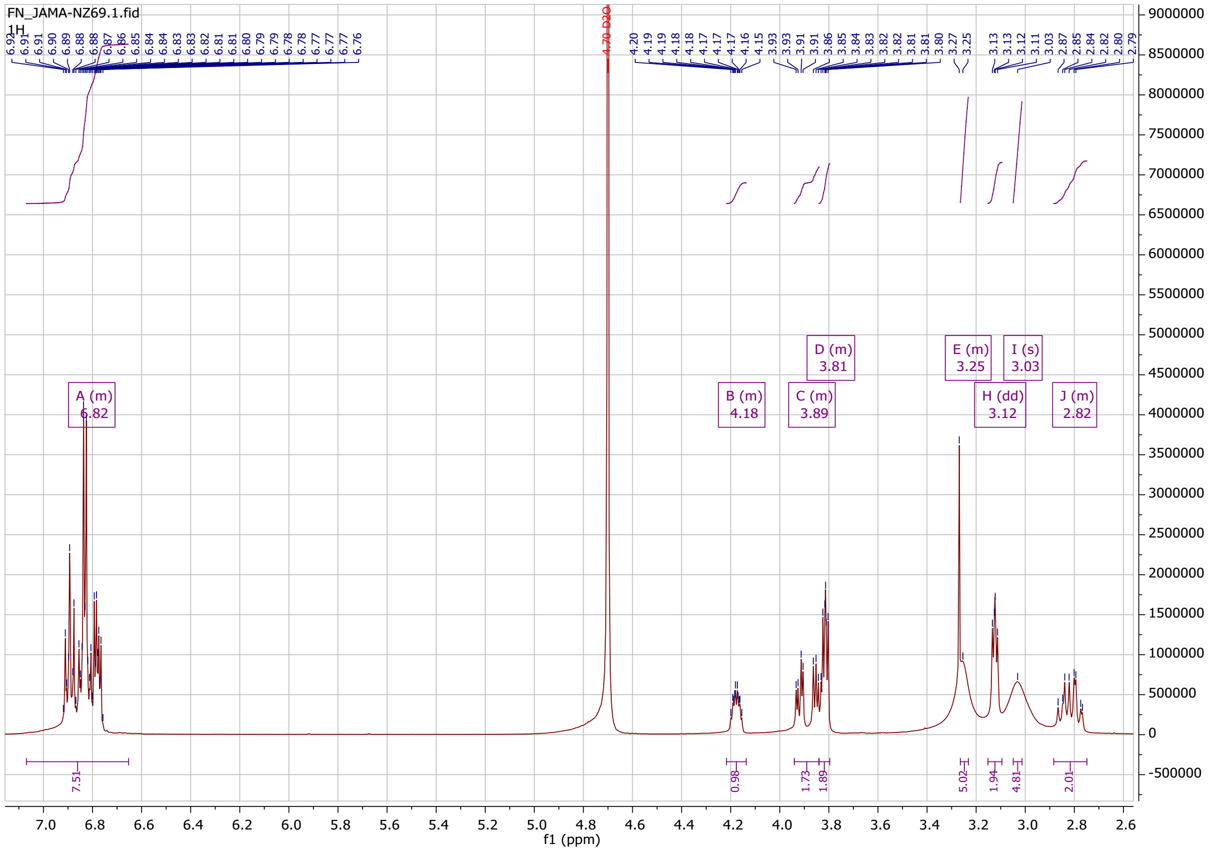

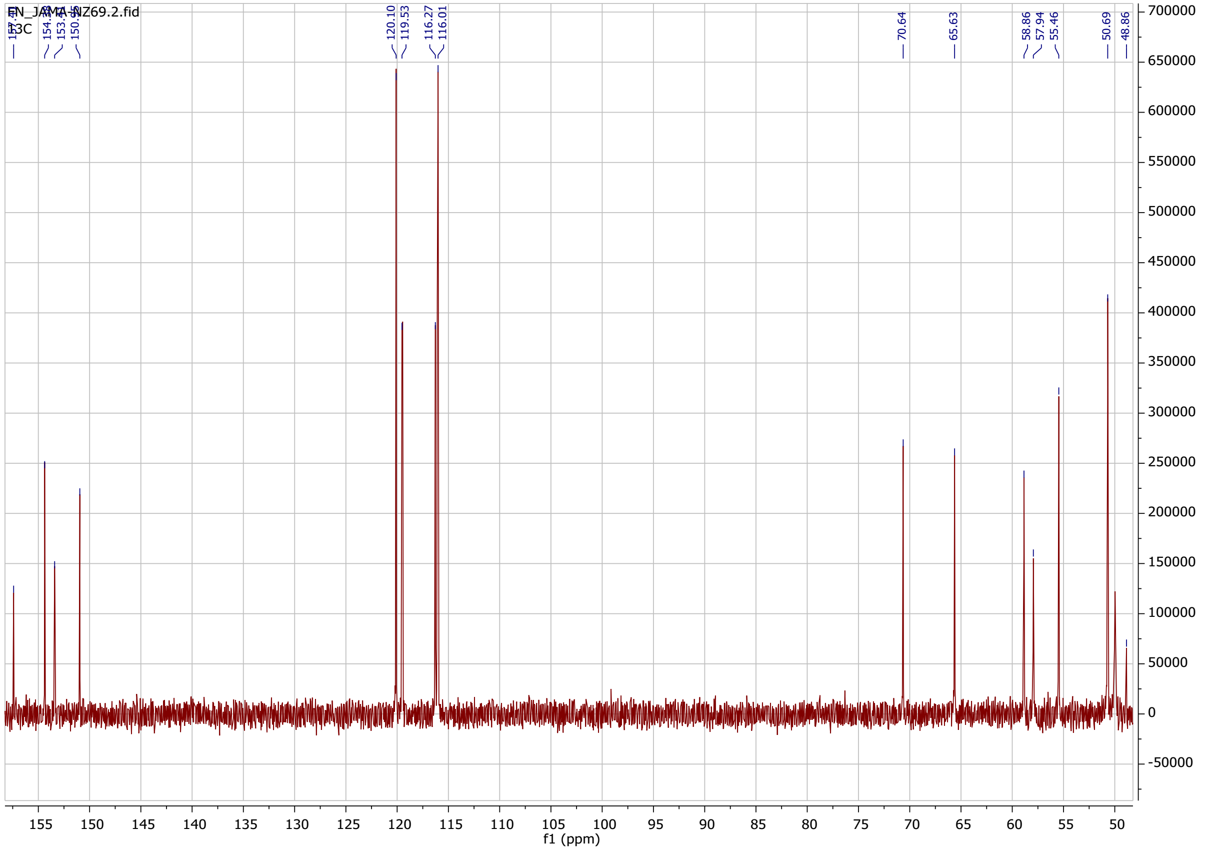
FN_JAMA-NZ69.2.fid
13C
120.10
119.53
116.27
116.01
70.64
65.63
58.86
57.94
55.46
50.69
48.86
f1 (ppm)

NZ-58_JM12 #275 RT: 2.90 AV: 1 NL: 5.77E9
T: FTMS + p ESI Full ms [105.0000-1000.0000]

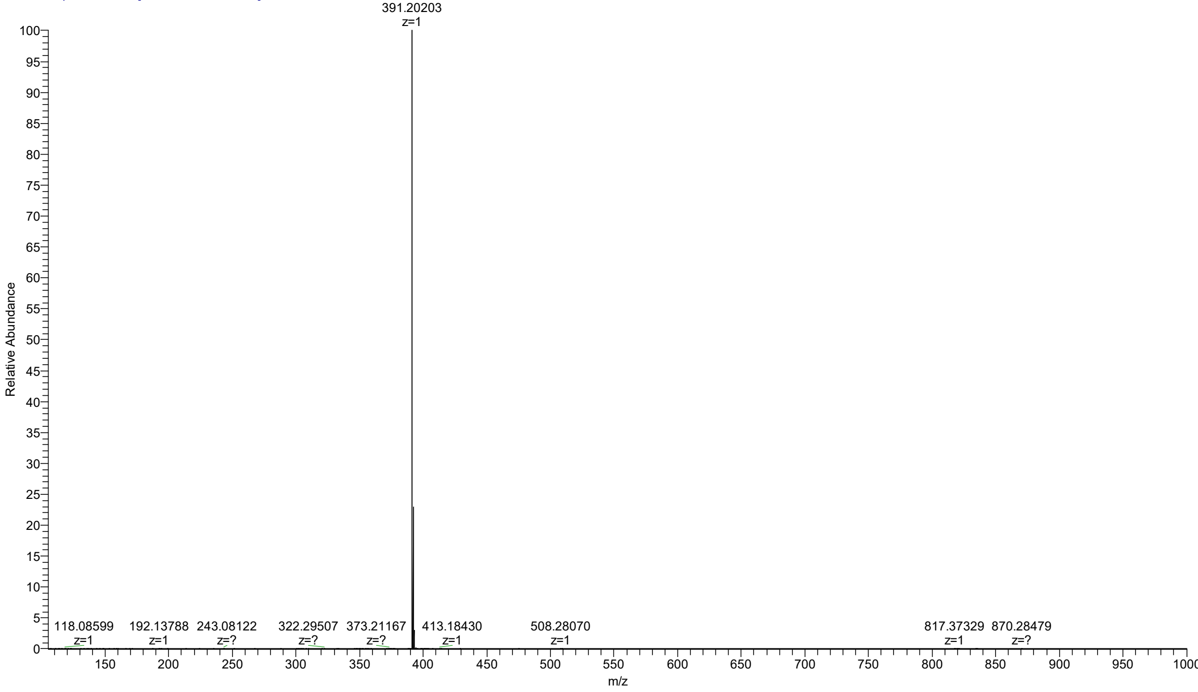

1-(4-(2-hydroxyethyl)piperazin-1-yl)-3-(2,4,5-trichlorophenoxy)propan-2-ol (**8**)

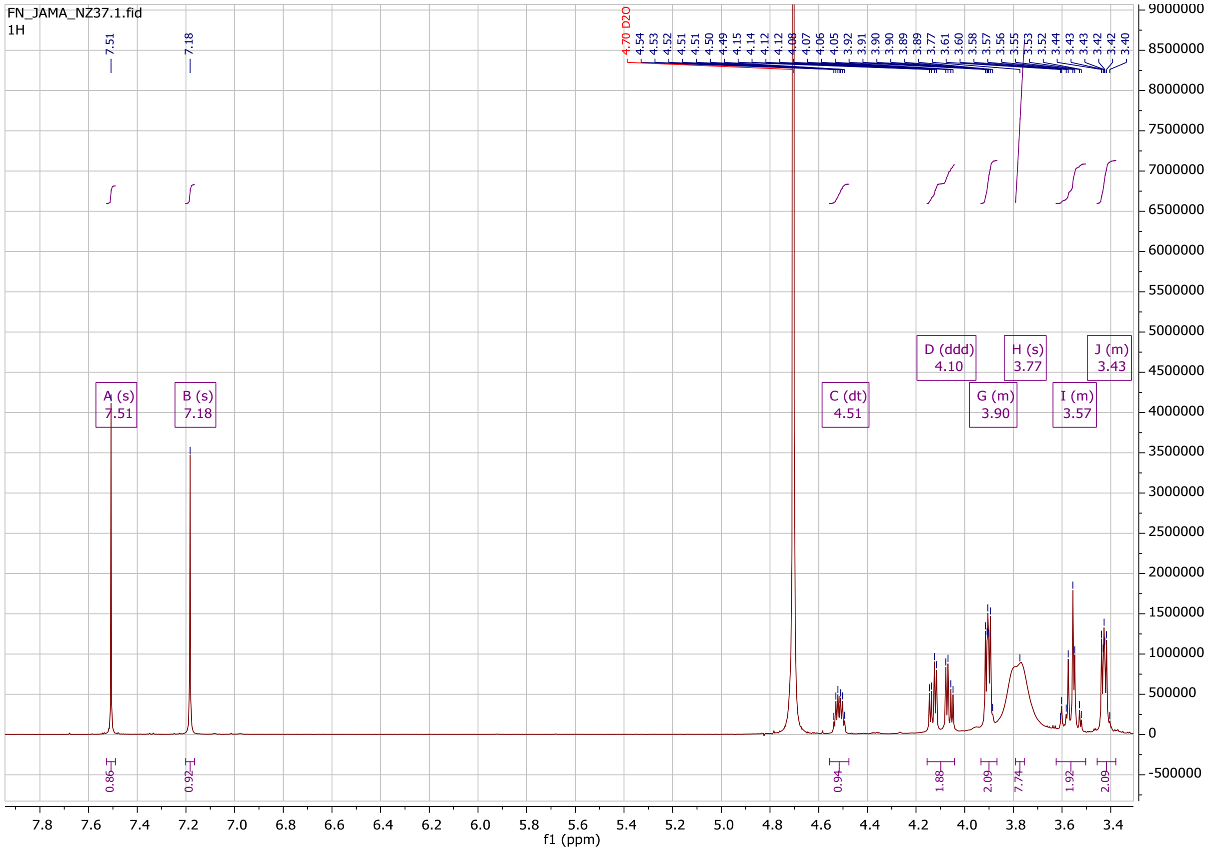

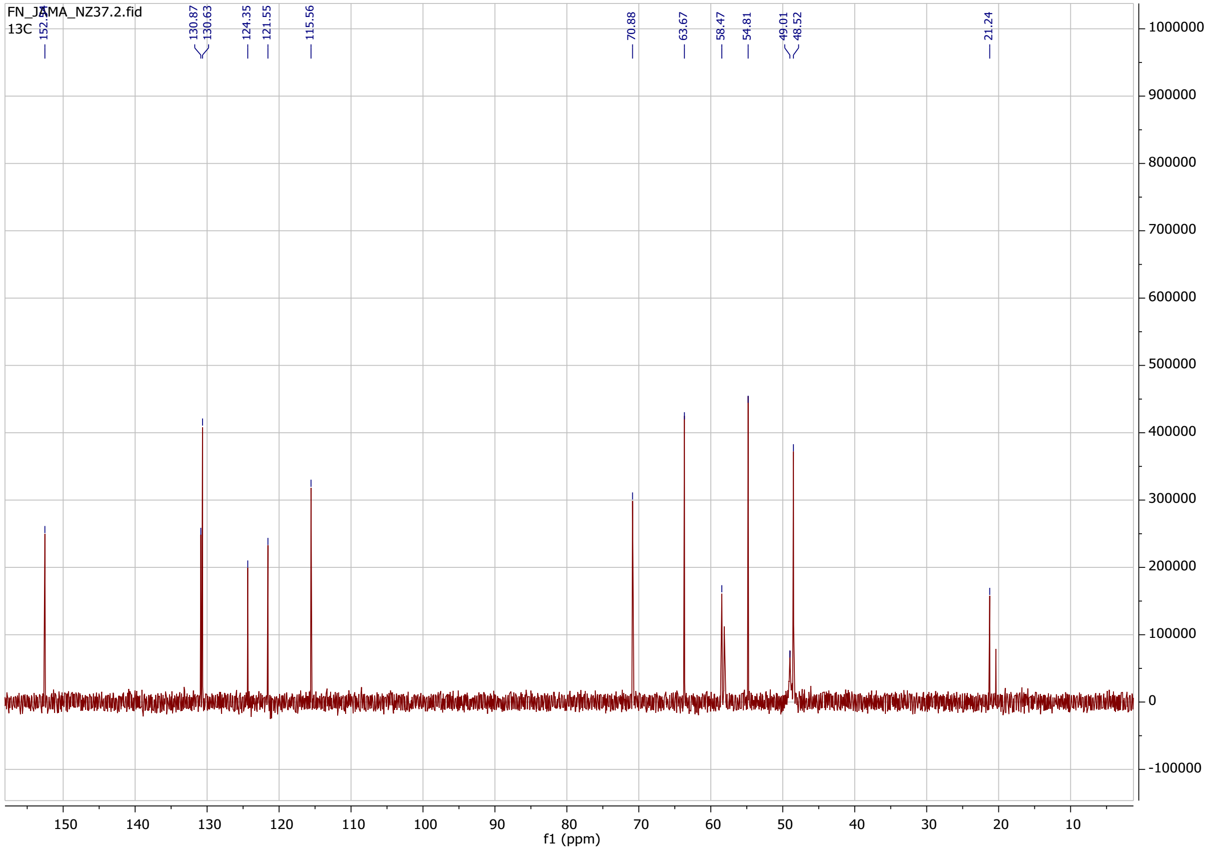

FN_JAMA_NZ37.2.fid
13C
152.5
130.87
130.63
124.35
121.55
115.56
70.88
63.67
58.47
54.81
49.01
48.52
21.24
f1 (ppm)

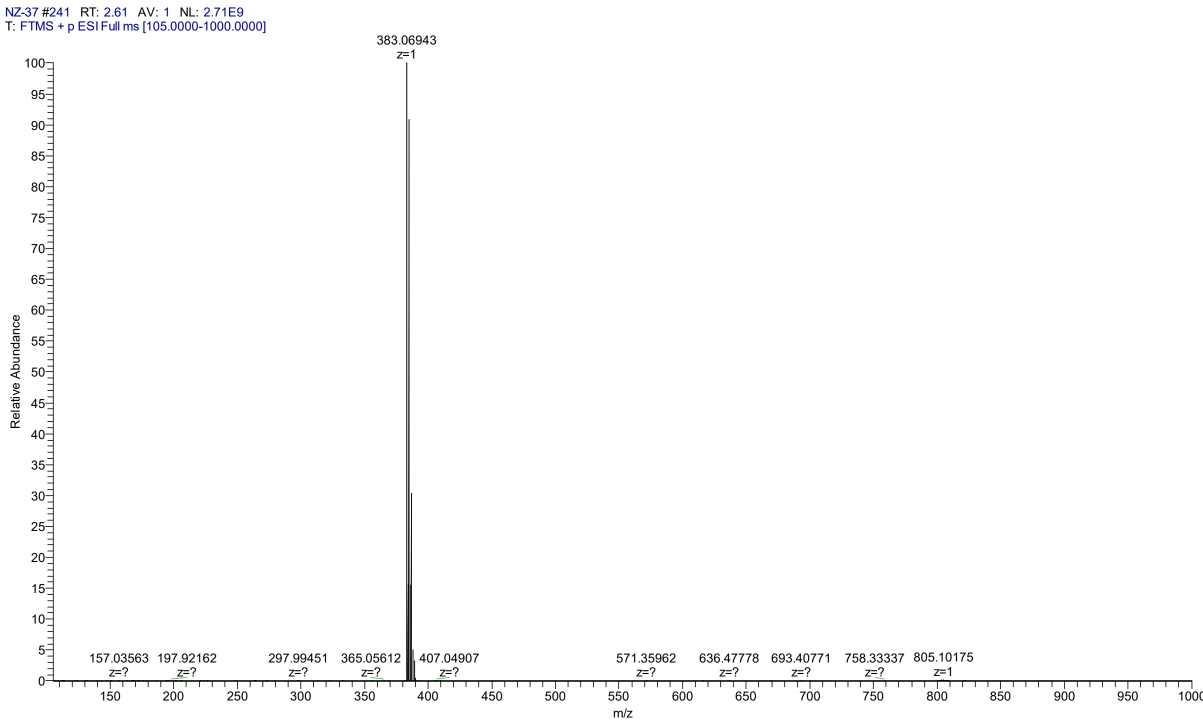
NZ-37 #241 RT: 2.61 AV: 1 NL: 2.71E9
T: FTMS + p ESI Full ms [105.0000-1000.0000]
383.06943
z=1
157.03563
z=?
197.92162
z=?
297.99451
z=?
365.05612
z=?
407.04907
z=?
571.35962
z=?
636.47778
z=?
693.40771
z=?
758.33337
z=?
805.10175
z=1
Relative Abundance
m/z
